# Chronic activation of anti‐oxidant pathways and iron accumulation in epileptogenic malformations

**DOI:** 10.1111/nan.12596

**Published:** 2020-01-14

**Authors:** T. S. Zimmer, G. Ciriminna, A. Arena, J. J. Anink, A. Korotkov, F. E. Jansen, W. van Hecke, W. G. Spliet, P. C. van Rijen, J. C. Baayen, S. Idema, N. R. Rensing, M. Wong, J. D. Mills, E. A. van Vliet, E. Aronica

**Affiliations:** ^1^ Department of (Neuro)Pathology Amsterdam UMC University of Amsterdam Amsterdam Neuroscience Amsterdam The Netherlands; ^2^ Department of Biochemical Sciences Sapienza University of Rome Rome Italy; ^3^ Department of Paediatric Neurology University Medical Center Utrecht The Netherlands; ^4^ Department of Pathology University Medical Center Utrecht The Netherlands; ^5^ Department of Neurosurgery Brain Centre Rudolf Magnus Institute for Neuroscience University Medical Center Utrecht Utrecht The Netherlands; ^6^ Department of Neurosurgery Amsterdam UMC Vrije Universiteit Amsterdam Amsterdam Neuroscience Amsterdam The Netherlands; ^7^ Department of Neurology Washington University Saint Louis MO USA; ^8^ Center for Neuroscience Swammerdam Institute for Life Sciences University of Amsterdam Amsterdam The Netherlands; ^9^ Stichting Epilepsie Instellingen Nederland (SEIN) Heemstede The Netherlands

**Keywords:** epilepsy, focal cortical dysplasia, haem oxygenase 1, iron metabolism, oxidative stress, tuberous sclerosis complex

## Abstract

**Aims:**

Oxidative stress is evident in resected epileptogenic brain tissue of patients with developmental brain malformations related to mammalian target of rapamycin activation: tuberous sclerosis complex (TSC) and focal cortical dysplasia type IIb (FCD IIb). Whether chronic activation of anti‐oxidant pathways is beneficial or contributes to pathology is not clear.

**Methods:**

We investigated oxidative stress markers, including haem oxygenase 1, ferritin and the inflammation associated microRNA‐155 in surgically resected epileptogenic brain tissue of TSC (*n* = 10) and FCD IIb (*n* = 8) patients and in a TSC model (*Tsc1* ^GFAP−/−^ mice) using immunohistochemistry, *in situ* hybridization, real‐time quantitative PCR and immunoblotting. Using human foetal astrocytes we performed an *in vitro* characterization of the anti‐oxidant response to acute and chronic oxidative stress and evaluated overexpression of the disease‐relevant pro‐inflammatory microRNA‐155.

**Results:**

Resected TSC or FCD IIb tissue displayed higher expression of oxidative stress markers and microRNA‐155. *Tsc1* ^GFAP−/−^ mice expressed more microRNA‐155 and haem oxygenase 1 in the brain compared to wild‐type, preceding the typical development of spontaneous seizures in these animals. *In vitro*, chronic microRNA‐155 overexpression induced haem oxygenase 1, iron regulatory elements and increased susceptibility to oxidative stress. Overexpression of iron regulatory genes was also detected in patients with TSC, FCD IIb and *Tsc1* ^GFAP−/−^ mice.

**Conclusion:**

Our results demonstrate that early and sustained activation of anti‐oxidant signalling and dysregulation of iron metabolism are a pathological hallmark of FCD IIb and TSC. Our findings suggest novel therapeutic strategies aimed at controlling the pathological link between both processes.

## Introduction

A hallmark seen in resected brain tissue of patients suffering from acquired forms of epilepsy, such as temporal lobe epilepsy (TLE), is the excessive generation of reactive oxygen species (ROS) that leads to oxidative stress (OS) 1, 2, 3. Recently, we showed for the first time strong expression of OS markers, in particular the light chain and catalytic cysteine/glutamate antiporter of the x_c_ system (SLC7A11 referred to as xCT), in resected brain tissue of patients suffering from epileptogenic developmental malformations, namely focal cortical dysplasia (FCD) type IIb and tuberous sclerosis complex (TSC) 4. Histopathological hallmarks of both pathologies include cortical dyslamination, presence of dysmorphic neurones and large, improperly developed immature cells called balloon cells in FCD IIb or giant cells in TSC 5, 6. Both pathologies represent mTORopathies, characterized by increased cellular mammalian target of rapamycin (mTOR) activation due to mutations in genes in this signalling pathway, which leads to the generation of brain lesions and epileptogenesis 7. Despite advances in understanding the contribution of mTOR hyperactivation to the pathogenesis of TSC and FCD IIb and initial clinical trials of mTOR inhibitors in TSC 8, 9, 10, more specific pathogenic alterations that could be targeted by pharmacotherapy remain to be identified. More specifically, only a subset of cells display mTOR hyperactivation making cell‐specific therapeutics preferable over broad‐spectrum mTOR inhibitors, particularly during brain development.

Prior studies demonstrated that overexpression of an essential enzyme in the synthesis of the primary intracellular anti‐oxidant glutathione, glutathione‐cysteine ligase catalytic subunit (GCLC), is required for redox adaptation and growth advantage of maldeveloped cells in TSC 11. Another study identified increased endoplasmic reticulum stress in TSC and indications for an increase in the activation of OS responsive genes 12. Collectively, these findings suggest that the cellular substrate for mTORopathies display increased OS coupled with increased OS resistance. Interestingly, expression of components of glutathione synthesis is directly regulated by the nuclear factor erythroid 2 like 2 (NFE2L2 referred to as Nrf‐2) transcription factor, a master regulator of the cellular response to OS 13, 14. Upon OS, Nrf‐2 activates many genes with cytoprotective roles, i.e. xCT and GCLC. Another important Nrf‐2 target is haem oxygenase 1 (HO‐1), an enzyme responsive to a variety of pro‐inflammatory and pro‐oxidant stimuli. It catabolizes potentially pro‐oxidant haem into radical scavenging and anti‐inflammatory bilirubin making it an important enzyme in first line cellular defence against OS and xenobiotics 15, 16, 17.

Since the presence of OS and overexpression of xCT and GCLC are consistent findings in TSC and FCD IIb we were interested if chronic activation of the Nrf‐2 pathway has protective or pathogenic implications. Additionally, we were interested in what disease‐relevant mechanisms could lead to sustained activity of this pathway. Here, we were particularly interested in microRNA‐155 (miR155) as it is upregulated in TLE and TSC, as well as in different rat models of epileptogenesis, all suggesting an important role for miR155 in epilepsy and epileptogenesis 18, 19, 20, 21. Moreover, miR155 was previously shown to be implicated in the regulation of OS in endothelial cells 22, 23.

The aim of this study was to investigate cellular damage due to OS and Nrf‐2 activation in TSC and FCD IIb and elucidate the potential contribution of miR155. Moreover, we were interested in changes in iron metabolism due to its potentially pathogenic interaction with ROS and because mTOR is a master regulator of a variety of metabolic processes. To this extent, we used surgically resected brain tissue from patients with drug‐resistant epilepsy due to FCD IIb or TSC and a mouse epilepsy model based on conditional *Tsc1* deletion in glial fibrillary acidic protein (GFAP) expressing cells (*Tsc1* ^GFAP−/−^ mice) to investigate these interactions. Finally, we overexpressed miR155 *in vitro* in human foetal astrocytes to investigate the mechanistic link between the factors involved.

## Materials and methods

### Subjects

The cases included in this study were obtained from the archives of the departments of Neuropathology of the Amsterdam UMC (Amsterdam, the Netherlands) and the University Medical Center Utrecht (UMCU, Utrecht, the Netherlands). Cortical brain samples were obtained from patients undergoing surgery for drug‐resistant epilepsy and diagnosed with FCD IIb (*n* = 8) or TSC (*n* = 14). All cases were reviewed independently by two neuropathologists, and the diagnosis of FCD was confirmed according to the international consensus classification system proposed for grading FCD 5. All patients with cortical tubers fulfilled the diagnostic criteria for TSC 6. None of the FCD patients fulfilled the diagnostic criteria for TSC. Control material was obtained at autopsy from age‐matched controls, without a history of seizures or other neurological diseases. All autopsies were performed within 24 h after death. Tissue was obtained and used in accordance with the Declaration of Helsinki and the Amsterdam UMC Research Code provided by the Medical Ethics Committee. Clinical information about the patients can be found in Table S1. Details on immunohistochemistry and molecular analysis are described in the supplementary methods.

### *Tsc1* ^GFAP−/−^ mice

All animal experiments in this study were approved by the Washington University Animal Welfare committee. *Tsc1* ^flox/flox^‐GFAP‐Cre knockout (*Tsc1* ^GFAP−/−^) mice with conditional inactivation of the *Tsc1* gene in astrocytes and neurones were generated as described previously 24, 25. *Tsc1* ^flox/+^‐GFAP‐Cre and *Tsc1* ^flox/flox^ littermates have previously been found to have no abnormal phenotype and were used as controls in these experiments. Cortex and hippocampus were collected from 2‐week‐old and 2‐month‐old animals (*n* = 5 animals per group). These time points were chosen on the basis of a previous study in which the course of seizure development in this model was carefully analysed and it was concluded that earliest seizure onset is at 4 weeks of age with a dramatic progressive increase in seizure incidence and frequency at 6 weeks 26. Thus, to investigate the epileptogenic process at time points before and after the development of seizures in this model 2‐week‐old and 2‐month‐old *Tsc1* ^GFAP−/−^ mice were compared in this study. For quantitative real‐time polymerase chain reaction (PCR), tissues were mechanically minced and homogenized in Qiazol Lysis Reagent (Qiagen Benelux, Venlo, the Netherlands). Subsequent RNA isolation was done using the miRNeasy Mini kit (Qiagen Benelux, Venlo, the Netherlands) according to the manufacturer’s instructions. For immunohistochemistry, perfusion fixation was performed with 4% paraformaldehyde, brains were dissected and embedded in paraffin and processed as described in the supplementary methods.

### Transfection and stimulation of cell cultures

Human foetal astrocyte enriched cultures were established as described in the supplementary methods. Maximum tolerable doses of H_2_O_2_ and glucose oxidase (GO) were determined in foetal astrocyte‐enriched cultures with the 3‐(4,5‐dimethylthiazol‐2‐yl)‐2,5‐diphenyl tetrazolium bromide (MTT, Sigma‐Aldrich, St Louis, MO, USA) cell viability assay (Figure S1). Briefly, cells were treated with different concentrations of H_2_O_2_ (Sigma‐Aldrich, St Louis, MO, USA) or GO from *Aspergillus niger* (type II, Sigma‐Aldrich, St Louis, MO, USA) in complete culture medium for 3 h or 72 h respectively. To ensure maximum activity, stimulation medium containing GO was refreshed every 24 h. Subsequently, 0.5 mg/ml MTT reagent in complete medium was added to each well and cells were incubated for 1 h at 37°C and 5% CO_2_. Thereafter the reaction mixture was discarded and 100 μl acid isopropanol (4 mM HCl, 0.1% NP‐40 in isopropanol) was added to each well to stop colour development and solubilize cells. Cell density was determined by measuring spectrophotometric absorbance at 570 nm using a microplate reader (BMG Labtech, Ortenberg, Germany).

For transfection, cultures were transfected with either mimic negative control (Figure S1), miR155 mimic (Applied Biosystems, Carlsbad, CA, USA) or antisense miR155 LNA oligonucleotide (miR155 antagomiR, Ribotask ApS, Odense, Denmark) (Table S2). Oligonucleotides were delivered to the cells using Lipofectamine® 2000 transfection reagent (Life Technologies, Grand Island, NY, USA) at a final concentration of 50 nM for a total of 24 h and subsequently stimulated with H_2_O_2_ (3 h) or GO (72 h) respectively. Before harvesting cells for quantitative real‐time PCR and western blot analysis, cells were washed twice with phosphate buffered saline.

### Statistical analysis

Statistical analysis of cell culture experiments was performed with GraphPad Prism software version 5.01 (Graphpad software Inc., La Jolla, CA, USA) using the nonparametric Mann–Whitney *U* test or, for multiple groups, the nonparametric Kruskal–Wallis test with correction for multiple comparisons (Dunn’s method). *P* < 0.05 was assumed to indicate a significant difference. Data are presented as box plots for human and mouse data and mean ± SEM for cell culture data. For RNA‐Seq data (Supplementary methods) a Mann–Whitney *U* test followed by a Benjamini‐Hochberg correction for multiple comparison test was carried out. An adjusted *P*‐value < 0.05 was assumed to indicate statistical significance.

#### Data availability statement

The data that support the findings of this study are available from the corresponding author, upon reasonable request.

## Results

### Oxidative stress damage, Nrf‐2 activation and miR155 expression in FCD IIb and TSC

**Figure 1:**
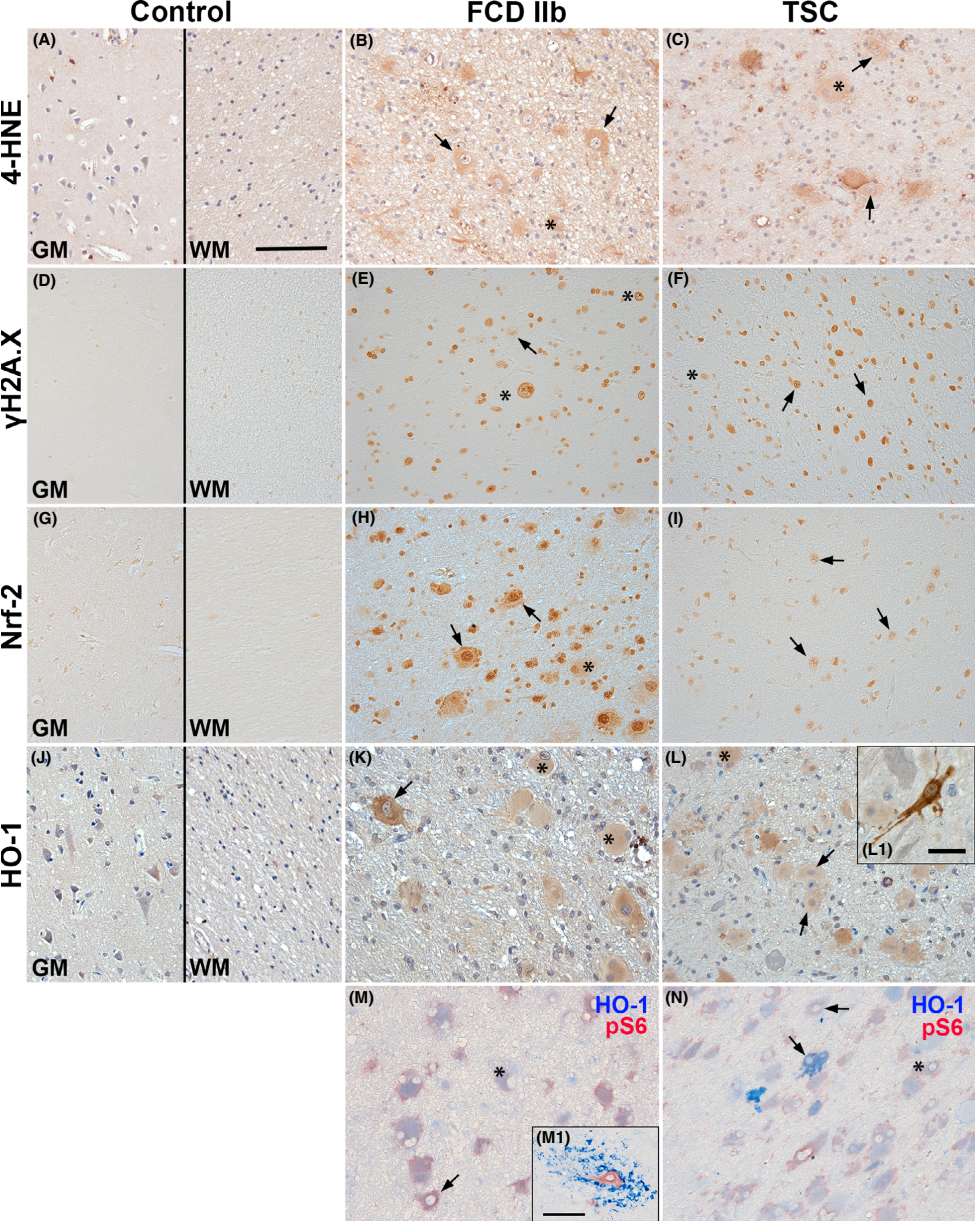
Higher expression of 4‐HNE, γH2A.X, Nrf‐2 and HO‐1 in TSC and FCD brain specimens. (**A, D, G, J**) Expression of OS markers in grey (GM) and white matter (WM) of autopsy control tissue. Low 4‐HNE and Nrf‐2 expression was observed in cells with neuronal and glial morphology in GM of control tissue, whereas γH2A.X was virtually absent in control tissue. HO‐1 was moderately expressed primarily in neurones in control GM and WM. (**B, C**) In FCD IIb and TSC 4‐HNE immunoreactivity was high in particular in dysmorphic neurones and balloon/giant cells, with higher reactivity in cellular processes surrounding dysmorphic neurones in TSC. (**E, F**) Expression of γH2A.X was much higher in all cell types in FCD IIb and TSC. (**H, I**) Nrf‐2 expression was higher in astrocytes and neurones in FCD IIb and TSC as compared to control, particularly in the nucleus. (**K, L**) HO‐1 reactivity was high in balloon/giant cells in FCD IIb and TSC tissue, with very high expression in dysmorphic neurones. (**M, N**) Double‐labelling with HO‐1 revealed consistent co‐localization with pS6 in FCD IIb and TSC, while some pS6‐positive dysmorphic neurones lacked HO‐1 expression (**M_1_**). High HO‐1 expression was predominately found in dysmorphic cells (**L_1_**). One FCD IIb case displayed cells with microglial morphology with very high HO‐1 reactivity surrounding dysmorphic neurones (**M_1_**). Sections A‐C and J‐L were counterstained with haematoxylin, Scale bars: 100 µm in A (representative for A–N), 20 µm in L_1_ and 100 µm in M_1_; arrows = dysmorphic neurones, asterisk = balloon/giant cells. [Colour figure can be viewed at wileyonlinelibrary.com]

To prove the presence of OS damage and activation of Nrf‐2 signalling in the epileptogenic pathologies FCD IIb and TSC, we assessed the expression of the OS damage marker 4‐hydroxynonenal (4‐HNE), the DNA damage marker phosphorylated H2A histone family member X (γH2A.X), as well as the expression of Nrf‐2 and HO‐1. The immunoreactivity score was increased for all markers in FCD IIb and TSC compared to control (Table 1). For the evaluation of immunohistochemical staining in FCD IIb and TSC tissue, cellular morphology was classified as glia (small nucleus and small soma), dysmorphic neurones (big central nucleus with enlarged soma and sometimes nonpolarized neurites) and giant/balloon cells (enlarged cells with glassy cytoplasm and laterally displaced nucleus) (see also 5. Expression of 4‐HNE was observed in control brain tissue, mainly in cortical neurones and glia (Figure 1 **A**). 4‐HNE reactivity in FCD IIb and TSC was observed especially in dysmorphic neurones and cells with glial morphology, but also in balloon/giant cells (Figure 1 **B,C**). Moreover, all cell types showed nuclear γH2A.X reactivity in FCD IIb and TSC tissue as compared to control where it was virtually absent (Figure 1 **D–F; **Figure S2). Nrf‐2 expression was found in cortical neurones and glia in control tissue, while expression in FCD IIb and TSC was located predominantly in the nucleus, but also in the cytoplasm of dysmorphic neurones and balloon/giant cells (Figure 1 **G–I**). HO‐1 expression was detectable in control tissue in neurones and glial cells. In FCD IIb and TSC tissue, HO‐1 expression was high in dysmorphic neurones and balloon/giant cells (Figure 1 **J–L**). In particular, sparsely distributed dysmorphic neurones displayed very strong reactivity (Figure 1 **K, L1**). To assess whether HO‐1 expression was high in cells with mTOR activation (indicated by pS6 expression) we performed double‐labelling with pS6 and HO‐1. This revealed HO‐1 expression in pS6‐positive dysmorphic neurones and balloon/giant cells in FCD IIb and TSC, with strong expression in particular subsets of cells as seen in single labelling (Figure 1 **M,N**). In addition, HO‐1 was also expressed in cells with microglial morphology in one case of FCD IIb (Figure 1 **M_1_**). Perilesional staining displayed a similar expression pattern as seen in autoptic control tissue, except for Nrf‐2 which was higher (Figure S3).

**Table 1:** Immunoreactivity score (IRS) for 4‐HNE, γH2A.X, Nrf‐2 and HO‐1 in surgically resected FCD IIb and TSC brain tissue

	Control	FCD IIb	TSC
4‐HNE	1 (1)	5 (4–6)*	4 (4)
yH2A.X	2 (1–4)	6 (4–9)*	6 (4–9)*
Nrf‐2	3 (3–6)	6 (6–9)*	6 (3–9)
HO‐1	2 (1–3)	2.5 (2–4)	3 (1–3)

The immunoreactivity score (IRS) is given as median with the range in brackets. Immunoreactivity was evaluated using a 0–3 scale (0 = absent, 1 = weak, 2 = moderate, 3 = strong staining). In addition, the number of positive cells was evaluated 0 = absent, 1 = rare, 2 = sparse, 3 = high). The product of these two scores (IRS) was calculated for each case. *n* = 5 (FCD IIb, TSC). Kruskal–Wallis test followed by the Dunn’s post hoc test; *P < 0.05 *vs.* control.

**Figure 2:**
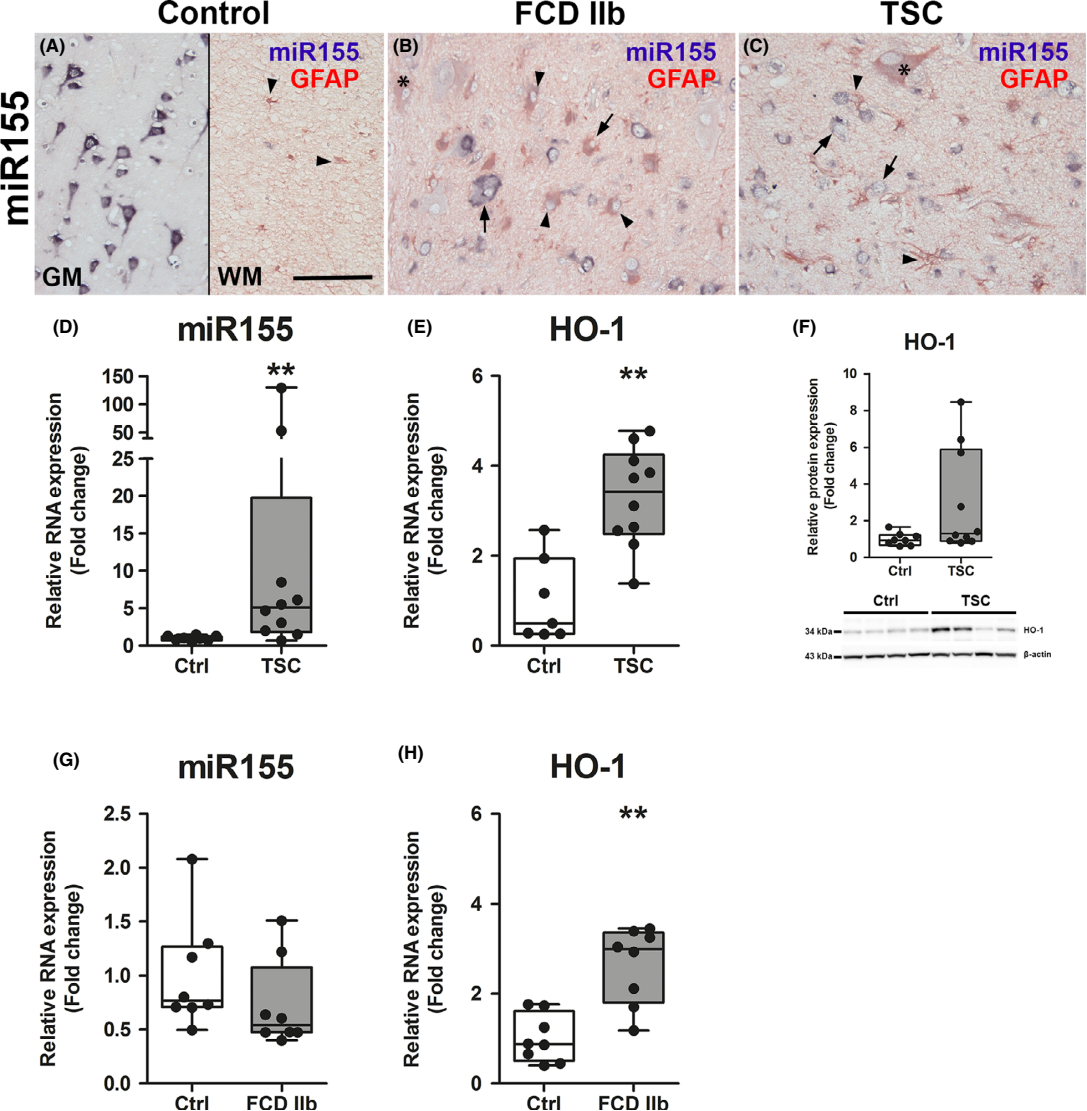
Higher expression of miR155 and HO‐1 in TSC and FCD IIb. (**A**) Expression of miR155 was predominately found in neurones of the grey matter (GM) with low expression in glia of the white matter (WM) (arrowheads). (**B, C**) In contrast, miR155 expression was predominantly found in GFAP‐positive cells with glial morphology and giant/balloon cells in FCD IIb and TSC as compared to control, whereas expression in dysmorphic neurones did not differ from control. (**D**) Expression of total miR155 in TSC tissue was higher than in control. (**E, F**) Moreover, HO‐1 RNA expression was higher in surgically resected tuber tissue from TSC patients compared to autoptic control, while HO‐1 protein expression was high only in a subset of patients. (**G, H**) miR155 expression was not different between autopsy control tissue and FCD IIb, while HO‐1 RNA was higher. Mann–Whitney *U* test. Data are expressed relative to expression observed in controls. Error bars represent range; **P < 0.01. *n* = 8 (Autopsy control, FCD IIb), *n* = 10 (TSC). Scale bar 100 µm in A (representative for A‐C), arrows = dysmorphic neurones, arrowheads = glia, asterisk = balloon/giant cells. [Colour figure can be viewed at wileyonlinelibrary.com]

In previous studies we already found that miR155 expression was higher in TSC tuber tissue as compared to control 19. Here, we wanted to verify this prior finding in an independent TSC cohort and also in a cohort of patients with FCD IIb. *In situ* hybridization revealed mainly neuronal expression of miR155 in the cortex of control tissue with occasional expression in GFAP‐positive cells in the WM (Figure 2 **A**). In FCD IIb and TSC tissue, miR155 expression in dysmorphic neurones was similar to that seen in neurones of control tissue. However, GFAP‐positive glial and balloon/giant cells appeared to have stronger miR155 expression as compared to GFAP‐positive cells in control tissue (Figure 2 **B,C** arrowheads). RNA quantification in surgically resected tissue homogenates of TSC patients confirmed stronger expression of miR155 as well as HO‐1 as compared to controls (Figure 2 **D–F**). HO‐1 protein was highly expressed in three out of 10 patients, while in seven patients the expression was similar to control (Figure 2 **F**, representative blot image). In FCD IIb, miR155 was not different to control (Figure 2 **G**). As in TSC, *HO‐1* expression was higher in patients with FCD IIb as compared to controls (Figure 2 **H**).

### *Tsc1* ^GFAP−/−^ mice display high 4‐HNE, HO‐1 and miR155 expression before the development of spontaneous seizures

**Figure 3:**
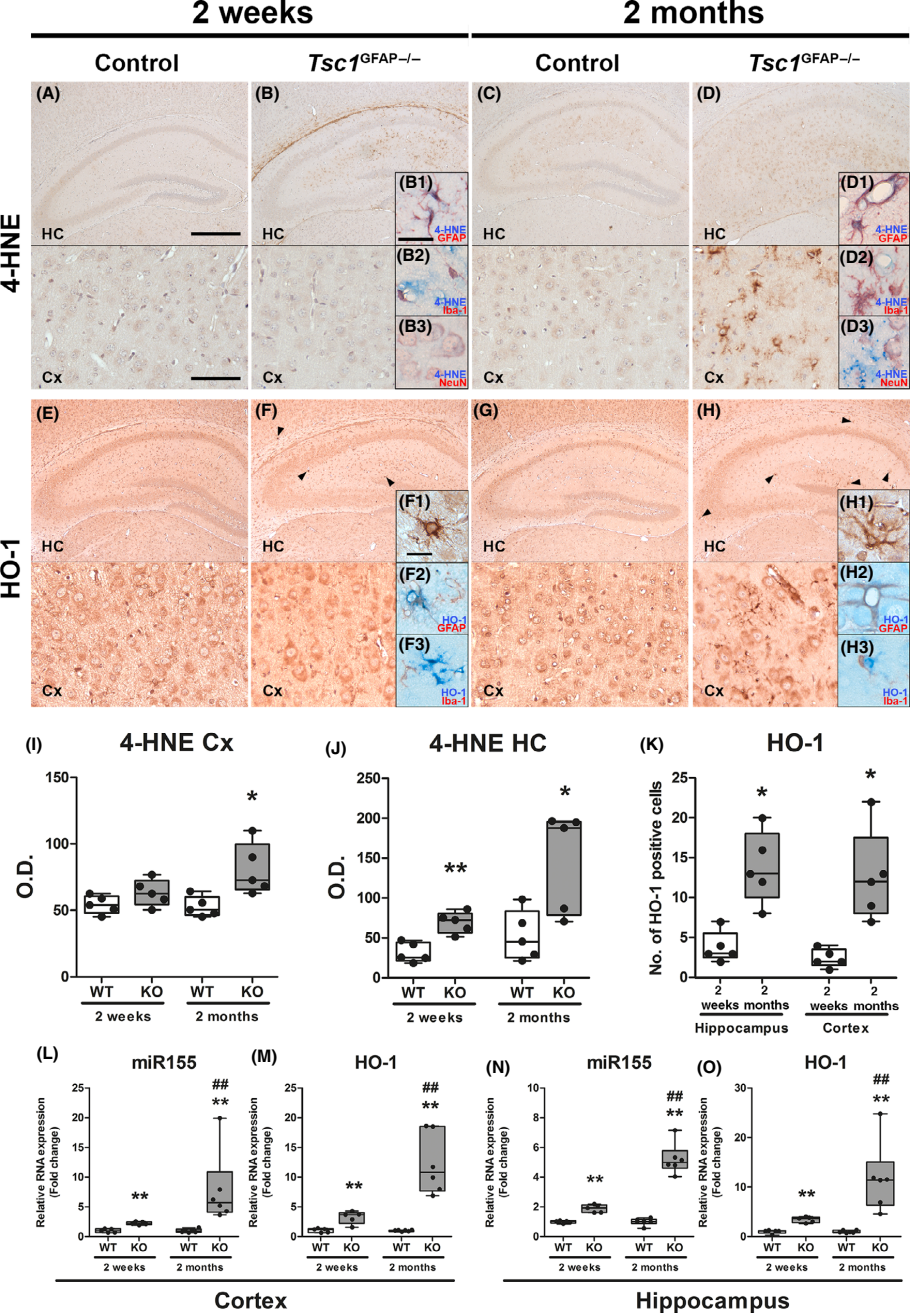
Higher expression of 4‐HNE, HO‐1 and miR155 in *Tsc1* ^GFAP−/−^ mice precedes the development of seizures. (**A, B**) Expression of 4‐HNE in the hippocampus (HC) and cortex (Cx) of 2‐week‐old control mice was low while it was detectable in 2‐week‐old *Tsc1* ^GFAP−/−^ mice before seizure onset, however, only in the hippocampus mainly perivascular and in GFAP and occasionally NeuN expressing cells, but not Iba‐1‐positive cells (**B_1_–B_3_**). (**C, D**) 4‐HNE expression was higher in the hippocampus of 2‐month‐old control mice after seizure onset compared to 2‐week‐old control mice before seizure onset. Two‐month‐old *Tsc1* ^GFAP−/−^ mice with recurrent seizures displayed high 4‐HNE expression in the hippocampus and cortex with similar perivascular expression and co‐localization with GFAP‐positive cells as in 2‐week‐old mice (**D_1_–D_3_**). (**E, G**) HO‐1 was moderately expressed in the hippocampus and cortex of 2‐week‐old and 2‐month‐old control mice. (**F**) In contrast, 2‐week‐old *Tsc1* ^GFAP−/−^ mice showed very high expression in sparsely distributed GFAP‐positive cells mainly in the hippocampus before seizure onset (arrowheads, **F_1_**). Iba‐1‐positive cells were in close proximity to the HO‐1 expressing cells, but did not show co‐localization (**F_2,3_**). (**H**) In 2‐month‐old *Tsc1* ^GFAP−/−^ mice with recurrent seizures the number of cells displaying strong HO‐1 expression was higher than in animals before seizure onset and could also be found in the cortex. (**I–J**) Quantification of 4‐HNE OD revealed higher 4‐HNE reactivity in 2‐week‐old animals in the HC and in HC and Cx in 2‐month‐old mice. (**K**) Additionally, the count of cells with strong HO‐1 expression increased in the HC and Cx in 2‐month‐old mice after seizure development (**F_1_, H_1_**). (**L–O**) RNA quantification of miR155 and HO‐1 in the hippocampus and cortex revealed higher expression in *Tsc1* ^GFAP−/−^ mice already before seizure onset compared to control, which was even higher in mice after the development of recurrent seizures. Scale bars: 500 µm (hippocampus) and 100 µm (cortex) in A, 50µm in B_1_ (representative of B_1_–B_3_, D_1_–D_3_) and 20 µm in insert in F_1_ (representative of F_1_–F_3_, H_1_–H_3_). Mann–Whitney *U* test. Data are expressed relative to expression observed in WT for the respective age group and presented as individual data points as well as in box plots. Error bars represent range; *P < 0.05, **P < 0.01 (L‐O ^##^ *P* < 0.01 2‐week‐old *vs.* 2‐month‐old *Tsc1* ^GFAP−/−^ mice). *n* = 5 animals per group. [Colour figure can be viewed at wileyonlinelibrary.com]

To investigate if mTOR hyperactivation *per se* could lead to the expression of 4‐HNE, HO‐1 and miR155 we analysed the expression of these markers in *Tsc1* ^GFAP−/−^ mice. Moreover, we compared tissue from mice before and after the onset of seizures to investigate if the expression of these markers preceded seizure development. 4‐HNE expression in the hippocampus of control mice was generally low, with slightly higher expression in 2‐month‐old mice compared to 2‐week‐old animals (Figure 3 **A,C**). In contrast, 2‐week‐old *Tsc1* ^GFAP−/−^ mice displayed higher 4‐HNE expression in the hippocampus before the development of seizures (Figure 3 **B,I,J**) which was even stronger in the cortex of 2‐month‐old *Tsc1* ^GFAP−/−^ mice with recurrent seizures (Figure 3 **D,I,J**). Here, 4‐HNE reactivity was predominantly detected in GFAP‐positive cells and occasionally NeuN‐positive cells (Figure 3 **B_1_–B_3_,D_1_–D_3_**). HO‐1 expression could be detected in all cell types in the hippocampus and cortex of control mice and there was no difference in expression between 2‐week‐old and 2‐month‐old control animals (Figure 3 **E,G**). In contrast, HO‐1 expression was very high in a small number of cells with glial morphology before seizure development (Figure 3 **F**, arrowheads and inserts F_1_, H_1_). The number of cells displaying very strong HO‐1 reactivity was higher after the development of seizures in cortex and hippocampus (Figure 3 **F,H,K**). As only cells with glial morphology were strongly HO‐1 reactive we wanted to know if these cells were astrocytes (positive for GFAP) or microglia (positive for Iba‐1). Double‐labelling revealed that cells displaying very strong HO‐1 reactivity were GFAP‐, but not Iba‐1‐positive (Figure 3 **F_2_, F_3_, H_2_, H_3_**), however, Iba‐1‐positive cells were often in close proximity to cells expressing high HO‐1. RNA quantification revealed higher expression of miR155 and *HO‐1* in the hippocampus and cortex of *Tsc1* ^GFAP−/−^ mice compared to wild‐type before seizure development, which displayed an even higher fold change compared to control after seizure development (Figure 3 **L–O**).

### *In vitro* prolonged activation of HO‐1 can be induced by miR155 and promotes expression of iron response genes

**Figure 4:**
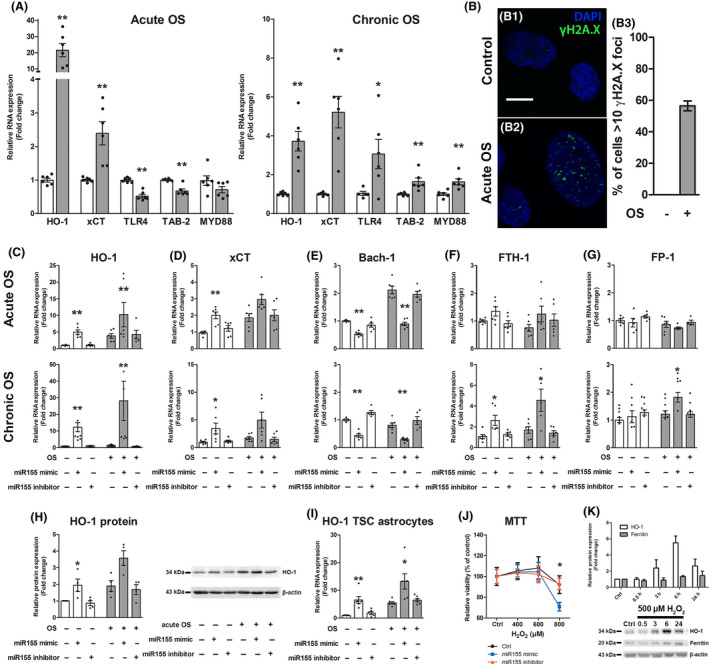
*In vitro*, human foetal astrocytes displayed different response to acute *vs.* chronic OS and chronic HO‐1 expression can induce genes involved in iron regulation. (**A**) Acute OS rapidly induced gene expression of Nrf‐2 targets HO‐1 and xCT, while NF‐κB signalling genes TLR‐4 and TAB‐2 were downregulated. Chronic OS increased expression of HO‐1 and xCT. However, the HO‐1 increase was lower than after acute OS. Moreover, in contrast to acute OS TLR‐4, TAB‐2 and MYD88 expression was increased. (**B**) Exposure to acute OS induced rapid expression of γH2A.X in the nucleus of human foetal astrocytes compared to control (B_2_). Quantification of cells displaying >10 γH2A.X foci could not be detected in control cells, while acute OS induced expression in approximately 55% of cells (B_3_). (**C, D, H, I**) Transfection of foetal astrocytes with miR155 mimic induced expression of HO‐1 and xCT even in the absence of OS. This effect was preserved in cells derived from TSC patients and could be reversed by the inhibitor of miR155. (**E**) Additionally, Bach‐1 expression was lower. These effects were independent of the presence of acute (3 h) or chronic (72 h) OS. (**F, G**) Transfection of foetal astrocytes with miR155 mimic for 24 h followed by acute OS had no effect on expression of FTH‐1 and FPN‐1. In contrast, prolonged exposure to the miR155 mimic for 72 h coupled to chronic OS induced FTH‐1 and FPN‐1. (**J**) Moreover, foetal astrocytes exposed to miR155 mimic for 72 h displayed increased susceptibility to high H_2_O_2_ concentrations compared to control and miR155 inhibitor transfected cells as measured using the MTT assay. (**K**) Foetal astrocytes stimulated with H_2_O_2_ for different time points displayed rapid expression of HO‐1 protein which peaked at 6 h and decreased again after 24 h. In parallel, ferritin expression increased with a delay and remained high even after 24 h. Scale bar is 10 µm in B. Mann–Whitney *U* test in A, J. Kruskal–Wallis test followed by Dunn’s in C–I, K. Data are expressed relative to expression observed in control groups and the mean value as well as the individual data points are shown. Error bars represent SEM; *P < 0.05, **P < 0.01, *n* = 3 independent cultures in duplicates (*n* = 379 cells (control) and 442 cells (acute OS) in B_3_; three single cultures for H, I, K; three independent cultures in quadruplicates for J) per experiment. [Colour figure can be viewed at wileyonlinelibrary.com]

We detected high HO‐1 expression in TSC, FCD IIb and *Tsc1* ^GFAP−/−^ mice. Thus we were interested whether HO‐1 is upregulated in both acute and prolonged OS and if this is accompanied by other pathogenic changes detected in TSC. Acute OS in human foetal astrocytes induced upregulation of *HO‐1* and *xCT* (Figure 4 **A**). In contrast, TLR4 signalling components *TLR4* and *TAB‐2* were downregulated while *MYD88* did not change. Chronic OS likewise induced *HO‐1* and *xCT* expression, however, the relative increase in *HO‐1* was less than in acute OS. Moreover, the expression of *TLR4*, *TAB‐2* and *MYD88* was higher in response to chronic OS (Figure 4 **A**). To prove that OS can induce DNA double strand damage as we detected in TSC and FCD IIb we investigated γH2A.X expression in foetal astrocytes and found a marked increase in response to acute OS (Figure 4 **B_1_–B_3_**).

Additionally, we were interested in miR155, which we showed previously to be upregulated and to contribute to the inflammatory phenotype in astrocytes. In particular, we wanted to know if miR155 could modulate OS‐reactive genes like HO‐1. Overexpression of miR155 in astrocytes led to higher expression of *HO‐1* and *xCT*, while the expression of BTB domain and CNC homolog 1 (*Bach‐1*), a putative miR155 target, was lower. These effects became stronger the longer cells were exposed to miR155 mimic but displayed only minor differences in the presence or absence of OS (Figure 4 **C–E**). In addition to RNA, the expression of HO‐1 protein was higher after miR155 transfection and this could also be replicated in cultured astrocytes derived from surgically resected tissue of TSC patients (Figure 4 **H,I**). Importantly, the expression was similar to controls when transfection with the miR155 inhibitor was performed. As we previously found miR155 to exert pro‐inflammatory effects, anti‐inflammatory HO‐1 activity could potentially alleviate these effects. However, HO‐1 activity also releases iron, which can potentially exacerbate OS by producing more reactive radicals via the Fenton reaction. Therefore, we analysed iron regulatory genes in response to miR155‐dependent chronic HO‐1 expression. Chronic, but not acute, exposure of human foetal astrocytes to miR155 induced expression of ferritin heavy chain 1 (*FTH‐1*) and *FP‐1* (Figure 4 **F,G**). Additionally, chronic exposure to miR155 made human foetal astrocytes more susceptible to a H_2_O_2_ challenge with a reduction to 70% viability compared to 90% in control and miR155 inhibitor transfected cells (Figure 4 **J)**. Finally, to investigate if endogenous HO‐1 expression in response to OS increases iron levels indicated by the surrogate marker ferritin, independent of miR155, we employed a time‐course of H_2_O_2_ stimulation. Foetal astrocytes exposed to H_2_O_2_ for different time points displayed rapid induction of HO‐1 protein expression 3 h after stimulation with maximum expression after 6 h and a decrease after 24 h. In parallel there was a slightly delayed ferritin induction after 6 h and it remained elevated even after 24 h (Figure 4 **K**).

### Increased ferritin expression and altered iron metabolism persists in FCD IIb, TSC and *Tsc1* ^GFAP−/−^ mice

**Figure 5:**
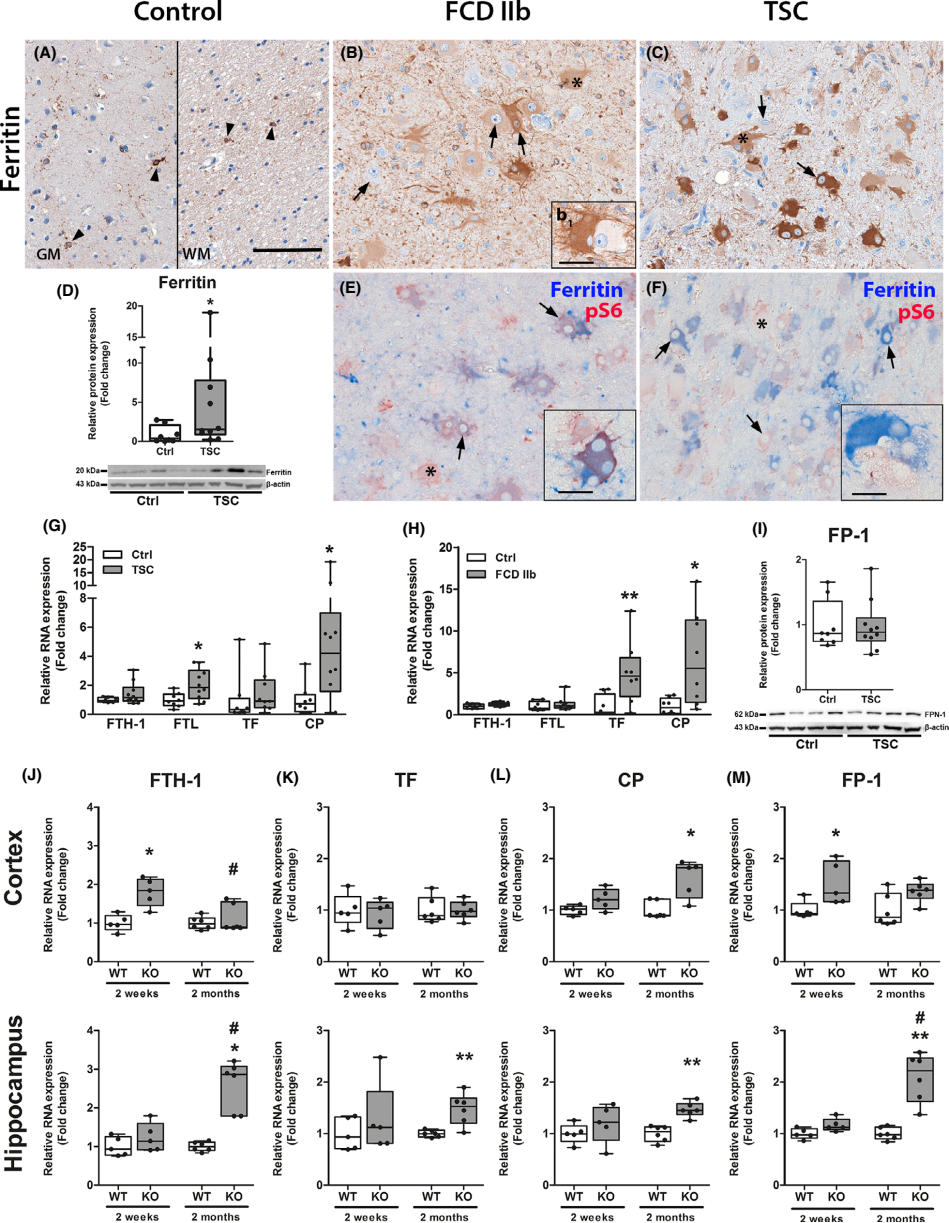
Ferritin expression and other markers of iron homeostasis were altered in TSC, FCD IIb and in *Tsc1* ^GFAP−/−^ mice. (**A**) Expression of ferritin in autoptic control tissue was restricted to cells with microglial morphology and very sporadically single cortical neurones (arrowheads). (**B, C**) In FCD IIb and TSC ferritin expression was high in the majority of dysmorphic neurones and balloon/giant cells. However, some cells displayed very weak ferritin expression (insert b_1_). (**D**) Consistent with immunohistochemical evaluation, western blot analysis showed that total ferritin protein was higher in TSC patients as compared to control. (**E, F**) Double‐labelling with pS6 revealed co‐localization with the majority of pS6‐positive cells in FCD IIb and TSC and additionally expression in cells with microglial morphology. Although most pS6‐positive cells co‐localized with ferritin some cells were negative for it (inserts E_1_, F_1_ red cells). (**G**) RNA expression revealed higher expression of FTL and CP in TSC tissue as compared to control. (**H**) In FCD IIb, expression of TF and CP was higher compared to controls. (**I**) The expression of the iron transporter FPN‐1 was not different between FCD IIb and TSC. (**J–M**) In the Cortex of *Tsc1* ^GFAP−/−^ mice expression of FTH1 and FP‐1 was higher before seizure onset, while CP was higher after seizure development. In the hippocampus *Tsc1* ^GFAP−/−^ mice displayed higher FTH1, TF, CP and FP‐1 expression after seizure onset. Scale bar 100 µm in A, 20 µm in inserts B_1_, E_1_, F_1_. Mann–Whitney *U* test. Data are expressed relative to expression observed in autopsy control tissue and control WT for the respective age group and presented as individual data points as well as in box plots. Error bars represent range; *P < 0.05, **P < 0.01. *n* = 5 animals per group (J–M ^#^ *P* < 0.05 2‐week‐old *vs.* 2‐month‐old *Tsc1* ^GFAP−/−^ mice). *n* = 8 (autopsy control, FCD IIb), *n* = 10 (TSC) (D, G–I.) [Colour figure can be viewed at wileyonlinelibrary.com]

As our *in vitro* data suggested that prolonged activation of anti‐oxidant pathways and HO‐1 activity induced ferritin, likely due to iron release, we investigated ferritin and other markers of iron metabolism in resected epileptogenic brain tissue of patients with FCD IIb, TSC as well as in *Tsc1* ^GFAP−/−^ mice. Ferritin expression in autopsy control brain tissue was found exclusively in cells with microglial morphology and was very rarely detected in neurones (Figure 5 **A**). In contrast, dysmorphic neurons and balloon/giant cells in FCD IIb and TSC displayed higher expression (Figure 5 **B,C**). Importantly, this high expression was found in most but not all cells, some of which were negative for ferritin (Figure 5 **B_1_**). Total ferritin protein was higher, however, similar to HO‐1 only a subset of four out of 10 cases had very high expression, whereas the rest showed moderately higher expression compared to control (Figure 5 **D**). Double‐labelling with pS6 showed consistent co‐localization with ferritin in most cells, however, pS6‐positive ferritin‐negative cells were also present in FCD IIb and TSC (Figure 5 **E,F,E_1_,F_1_** red cells). RNA expression revealed higher expression of ferritin light chain (*FTL*) and *CP* in TSC and *TF* and *CP* in FCD IIb (Figure 5 **G,H**). *FP‐1* protein expression was not changed in TSC (Figure 5 **I**). RNA expression in *Tsc1* ^GFAP−/−^ mice displayed higher expression of *FTH‐1* and *FP‐1* before typical seizure onset and higher *CP* expression after seizure onset in the cortex. In the hippocampus, *FTH‐1*, *TF*, *CP* and *FP‐1* expression was higher only after seizure onset (Figure 5 **J–M**).

### High 4‐HNE, HO‐1 and ferritin appear during TSC brain development and become cell‐specific in adolescent patients

**Figure 6:**
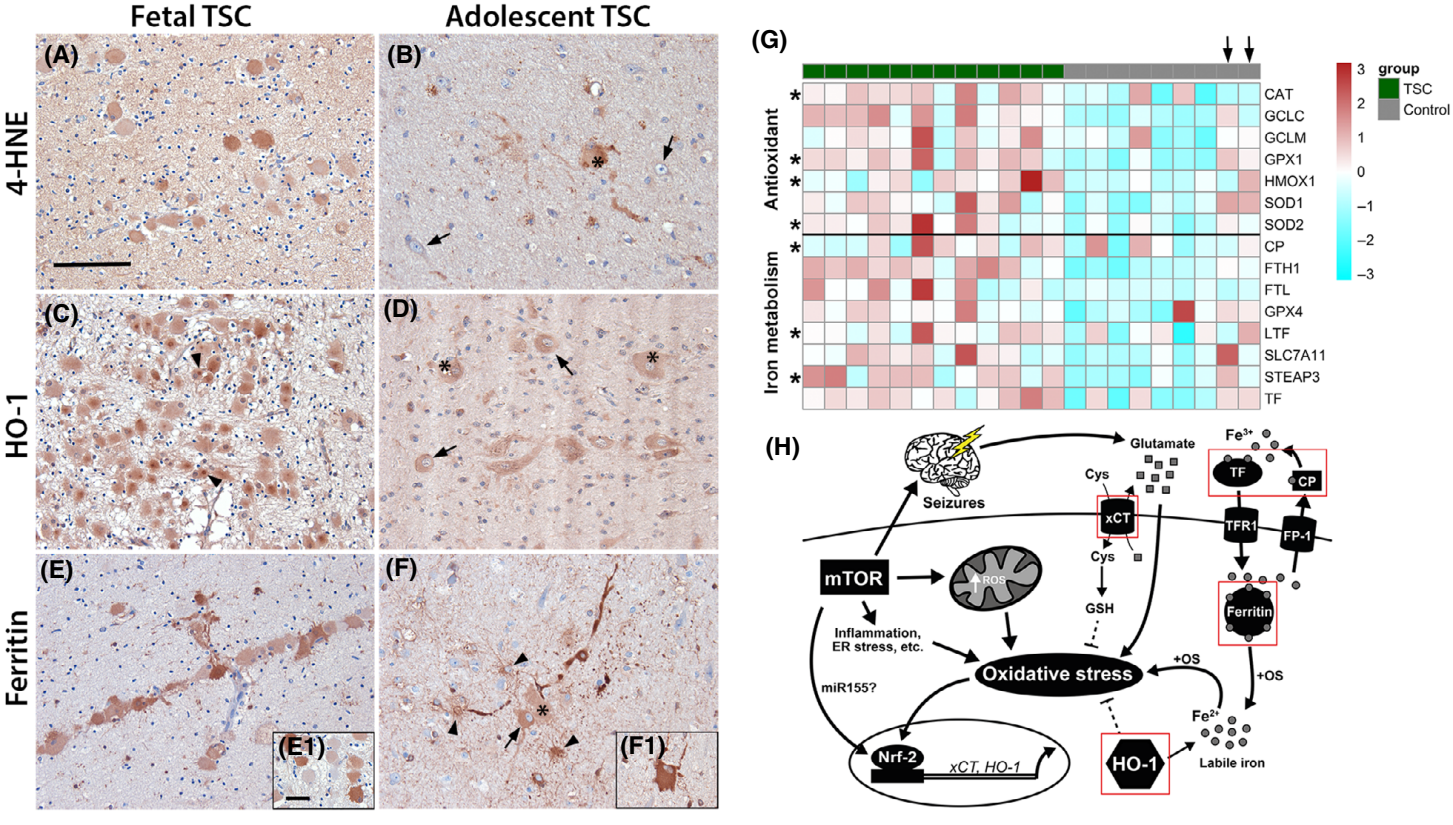
Chronic activation of OS response genes and altered iron metabolism preceded birth and persisted until adulthood. (**A**) Foetal TSC tissue displayed 4‐HNE reactivity in many immature cells with giant cell morphology. (**B**) Surgically resected TSC tissue of older patients displayed sporadic 4‐HNE reactivity predominantly in giant cells and cells with glial morphology but not dysmorphic neurones (arrows). (**C**) HO‐1 expression in foetal TSC brain was high in the majority of giant cells (arrows) with frequent highly HO‐1 reactive inclusions intracellularly next to the nucleus (arrowheads, insert in c). (**D**) In surgically resected TSC brain tissue of adolescent patients HO‐1 expression was high in dysmorphic neurones and giant cells (arrows, asterisks). (**E**) Ferritin expression in foetal brain tissue was markedly high in many but not all giant cells (insert E_1_). (**F**) In surgically resected brain tissue from older TSC patients cells displayed reactivity in dysmorphic neurones (arrows), giant cells (insert F_1_, asterisks) and cells with astrocytic morphology (arrowheads). (**G**) Heat map showing enrichment of anti‐oxidant genes and genes involved in iron metabolism in tubers from an independent cohort of TSC patients compared to autoptic control cortex. Please note the two control subjects indicated by an arrow on top that do show expression similar to the TSC cohort represent adult autopsy cases. Significant differences are indicated as asterisks in the respective row (Mann‐Whitney *U* test, BH‐adjusted probabilities *P* < 0.05). (**H**) Mechanisms of the interaction between mTOR activation and anti‐oxidant gene expression in the context of OS. Chronic activation of mTOR increases metabolic demand and ER stress, as well as inflammation, all inducing production of excess ROS leading to expression of anti‐oxidant genes, such as HO‐1 and xCT. Acute activation has protective effects (dashed lines), while sustained activation in turn contributes to OS via e.g. elevated extracellular glutamate via xCT or excess free iron via HO‐1. Disturbance in iron metabolism and seizures additionally contribute to free iron and glutamate and ultimately exacerbate OS even further. Components upregulated in TSC and FCD IIb are indicated by a red square. Sections A–C and J–L were counterstained with haematoxylin, Scale bar: 100 µm in A (representative for A–F), 20 µm in E_1_ (representative for inserts); arrows = dysmorphic neurones, arrowheads = inclusions in C, glia in F, asterisk = balloon/giant cells. [Colour figure can be viewed at wileyonlinelibrary.com]

We were interested if 4‐HNE, HO‐1 and ferritin expression affect cells during brain development in TSC. Moreover, we wanted to know how the expression pattern changes over time, thus we compared foetal TSC brain tissue to resected brain tissue from adolescent TSC patients. TSC tissue from foetal TSC brain (f, 27 gestational weeks (GW), TSC2 and f, 32 GW, TSC2) displayed 4‐HNE reactivity predominantly in foetal giant cells (Figure 6 **A**). In comparison, tubers of adolescent TSC patients (13 and 24 years old) displayed 4‐HNE reactivity in giant cells and cells with glial morphology, but only in a few dysmorphic neurones (Figure 6 **B**). Expression of HO‐1 was very high in giant cells in foetal TSC tissue displaying highly reactive intracellular inclusions (Figure 6 **C**, arrowheads). Lesions from adolescent TSC patients revealed the same HO‐1 expression pattern as seen in Figure 1 with high expression in dysmorphic neurones and giant cells (Figure 6 **D**). Ferritin expression in foetal TSC brain tissue was found in most neural precursor cells (Figure 6 **E**) with some displaying weak expression (Figure 6 **E_1_**). In resected brain tissue from adolescent TSC patients, ferritin expression was found in dysmorphic neurones, giant cells and cells with glial morphology (Figure 6 **F,F_1_**). Overall, expression of all three markers was already high in neural precursor cells during cell migration and proliferation (Figure 6 **C,E**). In the adolescent TSC brain, the expression remained high, but the number of positive cells was lower and became more cell type specific with increasing cellular differentiation (Figure 6 **F**). To generalize the dysregulation of anti‐oxidant genes and genes in iron metabolism in TSC we re‐analysed RNA sequencing data from an independent TSC cohort from a previous report 27. We found similar expression patterns for both pathways indicating a general upregulation of anti‐oxidant and iron regulatory genes (Figure 6 **G**). Interestingly, autoptic tissue from the only two old control subjects (39 and 44 years, (Figure 6 **G**, arrows) displayed activation of both pathways similar to TSC tissue, indicating an age‐dependent increase in healthy individuals.

## Discussion

We report evidence that TSC and FCD IIb are characterized by OS, chronic Nrf‐2 activation and provide indications that redox state and iron metabolism are altered in both these developmental malformations related to mTOR dysregulation. Moreover, our results suggest that these processes are present prior to seizure development and could contribute to epileptogenesis. Finally, our results imply that miR155 may contribute to Nrf‐2 activation and HO‐1 expression.

We previously identified that markers of OS are increased in TLE 3 as well as in malformations of cortical development 4. However, we did not investigate directly if malformations of cortical development also harbour cellular damage, which we prove here by showing higher lipid peroxidation and DNA double strand damage in TSC and FCD IIb brain tissue. Consistent with this, we see γH2A.X reactivity in acute OS *in vitro*. Interestingly, HO‐1 and 4‐HNE expression were highest in dysmorphic neurones and giant/balloon cells in TSC, FCD IIb and in astrocytes of *Tsc1* ^GFAP−/−^ mice. As mTOR activation persists in the brain of patients with TSC and FCD IIb and in astrocytes in *Tsc1* ^GFAP−/−^ mice it is interesting that only a subset of cells with mTOR activation display strong HO‐1 expression. This suggests that secondary mechanisms, other than only mTOR hyperactivity are necessary to induce sustained HO‐1 expression. One possible secondary mechanism could be the presence of positive modulators such as miR155. As miR155 expression can be induced by inflammation and immune responses its expression might also be driven by the same processes in TSC and FCD IIb 28. Importantly, we confirmed upregulation of miR155 in TSC tubers from a previous cohort 19 and show here for the first time higher miR155 expression prior to seizure development in *Tsc1* ^GFAP−/−^ mice. We could not detect higher expression in FCD IIb tissue, which might be due to cell type specific regulation or a lower fraction of cells with mTOR activation in FCD IIb lesions compared to TSC that cannot be detected on bulk tissue analysis. Mechanistically, *in vitro* human foetal astrocytes and TSC‐derived astrocytes transfected with miR155 show high expression of Nrf‐2 targets HO‐1 and xCT likely by miR155‐dependent inhibition of the Nrf‐2‐competitive transcription factor Bach‐1 29, 30. While miR155 can clearly modulate Nrf‐2 activity *in vitro*, we also believe that other factors in TSC and FCD IIb contribute to the expression of HO‐1, as it is an acute phase protein. This is also reflected by the discrepancy between HO‐1 RNA and protein expression on western blot indicating post‐transcriptional regulatory processes as shown previously 31. One example of post‐transcriptional control is miR155, which could have important implications in modulating sustained HO‐1 expression.

Nrf‐2 signalling and HO‐1 expression upon OS might help to defend the brain, resolving OS and inflammation. On the other hand, prolonged activity due to OS might be detrimental or contribute to pathogenesis in TSC and FCD IIb. To investigate the difference between acute and chronic Nrf‐2 activation we stimulated foetal astrocytes with H_2_O_2_ for 3 h or GO for 72 h *in vitro*. We observed HO‐1 upregulation upon OS in both conditions. However, while we measured a reduction in several genes of the pro‐inflammatory NF‐κB signalling pathway upon acute OS, likely via HO‐1 anti‐inflammatory action 32, 33, chronic OS led to higher expression of the very same genes. These results suggest a transient anti‐inflammatory effect of HO‐1 in response to pro‐oxidant stimuli that is replaced by secondary, pro‐inflammatory signalling cascades when chronically active 34. Therefore, we hypothesize that prolonged, excessive HO‐1 expression does not directly serve the anti‐inflammatory and anti‐oxidant purpose it does acutely. The potentially detrimental role of HO‐1 is supported by the increase in ferritin expression, a surrogate marker for intracellular iron concentrations, in foetal astrocytes chronically transfected with miR155. This effect likely results from haem‐derived free iron deposition via HO‐1. While we observed that HO‐1 expression is always accompanied by ferritin expression, likely to buffer released iron that otherwise participates in the Fenton reaction with H_2_O_2_ to produce even more reactive radicals, HO‐1 is rapidly suppressed again. However, cells with sustained miR155‐dependent HO‐1 expression are more susceptible to OS due to excess iron, leading to cell death. These observations are in agreement with studies showing that sustained HO‐1 activation can participate in oxidative modification of macromolecules via pro‐oxidant, labile iron 35, 36, 37, 38.

In addition to HO‐1, the Nrf‐2 target xCT was shown to be upregulated in glia, dysmorphic neurones and giant/balloon cells in TSC and FCD IIb 4. In this study we could show *in vitro* that miR155‐dependent Nrf‐2 stimulation also increases the expression of xCT. In the context of epilepsy and neuronal discharges increased extracellular glutamate provides an important determinant of xCT activity, which transports cysteine, the limiting amino acid in glutathione synthesis, in exchange for glutamate across membranes. In turn, high extracellular glutamate shifts the concentration gradient necessitating increased xCT expression for glutathione synthesis. Hence, cells expressing more xCT might be resistant to glutamate‐dependent excitotoxic OS. Intriguingly we see xCT overexpression in dysmorphic cells in TSC and FCD IIb, as well as in TLE 3 and these results synergize with previous reports showing that GCLC, another component of glutathione metabolism, is necessary for redox adaptation and aberrant cell growth in TSC2 mutant cells 11. Besides ferritin, we also detected specific RNA induction of the iron exporter *FP‐1* in foetal astrocytes chronically transfected with miR155, however, only in OS conditions. This suggests an increased necessity to secrete excess iron produced by HO‐1, specifically in pro‐oxidant conditions. In human TSC and FCD IIb brain tissue we detected higher RNA expression of ferritin, *TF* and *CP*, while FP‐1 protein expression was not changed. Although ferritin can sequester labile free iron, it was also shown that iron can be released again from ferritin in OS conditions 39, 40. The net effect of these changes suggests increased iron availability to cells, potentially further increasing the intracellular labile iron pool in addition to haem‐derived iron due to HO‐1 activity. Besides human tissue we also detected higher *FTH‐1*, *TF*, *CP* and *FP‐1* RNA in *Tsc1* ^GFAP−/−^ mice indicating changes in iron metabolism. Importantly, higher *FTH‐1* and *FP‐1* expression together with OS and HO‐1 expression precedes seizure development in the cortex of these mice. This is also seen in foetal TSC brain, in which neural precursor cells display dysregulated HO‐1 and ferritin expression together with 4‐HNE reactivity before birth and the formation of tubers. Importantly, we analysed an independent TSC cohort which supported upregulation of anti‐oxidant and iron regulatory genes, suggesting that this effect is a common phenomenon in TSC. All of these findings indicate a complex interconnection of mTOR, anti‐oxidant genes and iron regulatory genes, summarized in (Figure 6 **H**).

Considering high iron concentrations in the majority of dysmorphic cells in FCD IIb and TSC lesions, targeting these subsets of cells with ferroptotic agents might represent a promising novel treatment option. Ferroptosis is a regulated form of cell death due to iron‐dependent excessive lipid peroxidation 41. Here, HO‐1 was shown to be an essential component, with prolonged, excessive activation promoting cell death 42, 43, 44, 45. Ferroptosis‐mediated cell death ensues only when the intracellular defence mechanisms against lipid peroxidation and iron overload are overwhelmed. Key components in resistance to ferroptosis represent iron sequestering proteins like ferritin and proteins in thiol metabolism that provide the basis for anti‐oxidant glutathione synthesis, in particular xCT and glutathione peroxidase 4 (GPx4) which is important in detoxification of lipid peroxides and essential for neuronal survival 46, 47, 48. As prior studies verified increased expression of xCT and GCLC in TSC 4, 11, dysmorphic cells in TSC and FCD IIb likely increase Nrf‐2 activity to adapt to increased intracellular iron caused by mTOR activity, while paradoxically producing even more labile iron via HO‐1. While it was shown that increased mTOR activity makes cells more susceptible to apoptotic cell death 49, 50, ferroptosis might be another important contributor to cell loss during brain development in TSC patients. Indeed, we find evidence for prominent cell loss in postnatal tissue from FCD IIb and TSC subjects 51, 52. This suggests possible ongoing ferroptotic cell loss and redox adaptation, even after birth, further exacerbated by seizure activity. In essence, these processes could create a strong positive selection pressure for cells with resistance to OS and excess iron and indicate that pharmacotherapy targeting iron homeostasis or redox adaptation could be exploited to specifically target dysmorphic cells. Finally, it is generally believed that aberrant network formation in FCD IIb and TSC not only gives rise to epilepsy but also the frequently observed cognitive impairments 53, 54, 55. Interestingly, evidence from other pathologies like Alzheimer’s disease suggests that iron accumulation might act as a driver of cognitive deterioration by ferroptosis, OS or related inflammatory responses 56. Thus, disturbed iron homeostasis and iron overload could represent a common neuropathological mechanism underlying both, epilepsy and cognitive comorbidities in FCD IIb and TSC. We propose that therapeutics inhibiting redox or iron adaptation, such as Nrf‐2 inhibitors or ferroptotic agents, might have therapeutic value to specifically target a subset of pathogenic cells in TSC and FCD IIb. Additionally, this approach could circumvent side effects of current mTOR inhibitors. Moreover, these findings may have important implications in other selected subtypes of epilepsy with dysregulation of anti‐oxidant signalling and iron metabolism (such as TLE and other acquired focal epilepsies) and may yield new therapeutic strategies aimed at controlling the pathological link between oxidative stress, inflammation and the dysregulated iron metabolism, common neurobiological mechanisms underlying epilepsy and cognitive and behavioural comorbidities.

## Conflict of interest

The authors declare that they have no conflict of interest.

## Ethical approval

All procedures performed in studies involving human participants were in accordance with the Amsterdam UMC Research Code provided by the Medical Ethics Committee and with the 1964 Helsinki declaration and its later amendments or comparable ethical standards. All procedures performed in studies involving animals were in accordance with the ethical standards of the Washington University Animal Welfare committee.

## Supporting information


**Appendix S1. **Supplementary methods.
**Figure S1. **(**A**) The expression of miR155 after transfection in human foetal astrocytes was only marginally lowered after 72 h in chronic OS conditions. (**B**) Bach‐1 protein expression was lowered by miR155 in human foetal astrocytes transfected for 24 h. (**C**) Transfection of human foetal astrocytes with scrambled construct did not target Bach‐1 to induce HO‐1. (**D, E**) Human foetal astrocytes are decreased when exposed to >500 µM H_2_O_2_ (3 h) or >2.5 mU GO (24 h). Data in A‐C presented as mean, error bars represent SEM; *P < 0.05, **P < 0.01, ***P < 0.001. Data are representative of one (A) or three (B‐E) independent experiments with two (A‐C) or four (D, E) replicates for each group.
**Figure S2.** **(A, B) **Double labelling of Nrf‐2 with GFAP and NeuN revealed nuclear expression in astrocytes and neurones. (**C, D**) HO‐1 expression could be detected primarily in the cytoplasm of astrocytes, and some neurones, as well as in NeuN and GFAP negative cells. (**E, F**) Nuclear expression of γH2A.X could be detected in astrocytes and some neurones. Scale bar 100 µm in A, E.
**Figure S3. **(**A, B**) 4‐HNE reactivity in FCD IIb and TSC perilesional tissue was confined to low neuronal and perivascular expression. (**C, D**) γH2A.X expression in perilesional tissue could be detected in all cell types. (**E, F**) Perilesional Nrf‐2 expression was expressed in all cell types and higher than in autopsy control tissue. (**G, H**) HO‐1 expression in perilesional areas was mainly confined to neurones and some cells with glial morphology. Scale bar 100 µm in A.
**Table S1. **Clinical information of FCD IIb, TSC and autopsy control cases.
**Table S2. **Oligonucleotide sequence of miR155.
**Table S3. **Primer sequences used for quantitative real‐time PCR.
