# Supplementary material for: Chronic activation of anti‐oxidant pathways and iron accumulation in epileptogenic malformations
Source: Neuropathol Appl Neurobiol. 2020 Jan 14;46(6):546–63. doi: 10.1111/nan.12596 (PMC7308211; doi:10.1111/nan.12596)
Supplement: Supplementary file 1 — Appendix S1. Supplementary methods. Figure S1. (A) The expression of miR155 after transfection in human foetal astrocytes was only marginally lowered after 72 h in chronic OS conditions. (B) Bach‐1 protein expression was lowered by miR155 in human foetal astrocytes transfected for 24 h. (C) Transfection of human foetal astrocytes with scrambled construct did not target Bach‐1 to induce HO‐1. (D, E) Human foetal astrocytes are decreased when exposed to >500 µM H2O2 (3 h) or >2.5 mU GO (24 h). Data in A‐C presented as mean, error bars represent SEM; *P < 0.05, **P < 0.01, ***P < 0.001. Data are representative of one (A) or three (B‐E) independent experiments with two (A‐C) or four (D, E) replicates for each group. Figure S2. (A, B) Double labelling of Nrf‐2 with GFAP and NeuN revealed nuclear expression in astrocytes and neurones. (C, D) HO‐1 expression could be detected primarily in the cytoplasm of astrocytes, and some neurones, as well as in NeuN and GFAP negative cells. (E, F) Nuclear expression of γH2A.X could be detected in astrocytes and some neurones. Scale bar 100 µm in A, E. Figure S3. (A, B) 4‐HNE reactivity in FCD IIb and TSC perilesional tissue was confined to low neuronal and perivascular expression. (C, D) γH2A.X expression in perilesional tissue could be detected in all cell types. (E, F) Perilesional Nrf‐2 expression was expressed in all cell types and higher than in autopsy control tissue. (G, H) HO‐1 expression in perilesional areas was mainly confined to neurones and some cells with glial morphology. Scale bar 100 µm in A. Table S1. Clinical information of FCD IIb, TSC and autopsy control cases. Table S2. Oligonucleotide sequence of miR155. Table S3. Primer sequences used for quantitative real‐time PCR. [file NAN-46-546-s001.docx]

**Supplementary information**

**chronic activation of antioxidant PATHWAYS and iron accumulation in epileptogenic malformations**

**Till S. Zimmer^1^, Giulia Ciriminna^1^, Andrea Arena^1,2^, Jasper J. Anink^1^, Anatoly Korotkov^1^, Floor E. Jansen^3^, Wim van Hecke^4^, Wim G. Spliet^4^, Peter C. van Rijen^5^, Johannes C. Baayen^6^, Sander Idema^6^, Nicholas R. Rensing^7^ , Michael Wong^7^, James D. Mills^1^, Erwin A. van Vliet^1,8,*^, Eleonora Aronica^1,9,*^**

^1^Amsterdam UMC, University of Amsterdam, Department of (Neuro)Pathology, Amsterdam Neuroscience, Amsterdam, the Netherlands

^2^ Sapienza University of Rome, Department of Biochemical Sciences, Rome, Italy

^3^University Medical Center Utrecht, Department of Paediatric Neurology, Utrecht, the Netherlands

^4^University Medical Center Utrecht, Department of Pathology, Utrecht, the Netherlands

^5^University Medical Center Utrecht, Brain Centre, Rudolf Magnus Institute for Neuroscience, Department of Neurosurgery, Utrecht, the Netherlands
^6^Amsterdam UMC, Vrije Universiteit Amsterdam, Department of Neurosurgery, Amsterdam Neuroscience, Amsterdam, the Netherlands
^7^Washington University, Department of Neurology, Saint Louis, MO, United States of America

^8^University of Amsterdam, Swammerdam Institute for Life Sciences, Center for Neuroscience, Amsterdam, the Netherlands

^9^Stichting Epilepsie Instellingen Nederland (SEIN), Heemstede, the Netherlands

* these authors are joint senior author

**Supplementary Methods**

**Immunohistochemistry on human and mouse brain tissue**

Human brain tissue was fixed in 10 % buffered formalin and embedded in paraffin. Paraffin embedded tissue from human and mouse was sectioned at 5 µm, mounted on pre-coated glass slides (Star Frost, Waldemar Knittel Glasbearbeitungs, Braunschweig, Germany) and processed for immunohistochemical staining. Sections were deparaffinised in xylene, and ethanol (100 %, 95 %, 70 %) and incubated for 20 min in 0.3 % H_2_O_2_ diluted in methanol to block endogenous peroxidase activity. Antigen retrieval was performed using a pressure cooker in 0.01 M sodium citrate buffer (pH 6.0) at 121 °C for 10 min. Slides were washed with phosphate buffered saline (PBS; 0.1 M, pH 7.4) and incubated overnight with primary antibodies against phosphorylated H2A histone family member X (γH2A.X; mouse monoclonal, clone JBW301, Millipore, Darmstadt, Germany; 1:100), 4-hydroxynonenal (4-HNE; rabbit polyclonal, Abcam, Cambridge, UK; 1:500), Nrf-2 (rabbit polyclonal, Santa Cruz, Dallas, TX, USA; 1:500), HO-1 (rabbit polyclonal, Abcam, Cambridge, UK; 1:200) or ferritin (rabbit polyclonal, DAKO, Glostrup, Denmark; 1:750) in antibody diluent (VWR International, Radnor, PA, USA) at 4°C. Post-antibody blocking from the BrightVision+ system (containing rabbit anti-mouse IgG; Immunologic, Duiven, the Netherlands) was performed. Sections were washed in PBS and then stained with a polymer based peroxidase immunohistochemistry detection kit (Brightvision plus kit, ImmunoLogic, Duiven, the Netherlands) according to the manufacturer’s instructions. Staining was performed using Bright 3,3'-diaminobenzidine (DAB) substrate solution (ImmunoLogic, Duiven, the Netherlands). The reaction was stopped by washing in distilled water. Sections were counterstained with Haematoxylin-Mayer solution (Klinipath, Breda, the Netherlands), dehydrated in alcohol and xylene and coverslipped.

Double-labeling was performed with neuronal nuclear protein (NeuN; mouse monoclonal, clone MAB377; Chemicon, Temecula, CA, USA; 1:2,000), ionized calcium-binding adapter molecule 1 (Iba-1; rabbit polyclonal, WAKO, Osaka Japan, 1:2,000), glial fibrillary acidic protein (GFAP; mouse monoclonal, clone GA5, Sigma-Aldrich, St. Louis, MO, USA; 1:4,000) or phosphorylated S6 ribosomal protein (pS6; rabbit monoclonal, clone 91B2, Cell Signaling Technologies, Danvers, MA, USA; 1:100). Sections were incubated overnight with HO-1 or γH2A.X primary antibody and the next day incubated with BrightVision poly-alkaline phosphatase (AP) anti-rabbit (Immunologic, Duiven, the Netherlands) for 30 min at room temperature and washed with PBS. AP activity was visualized with the AP substrate kit III Vector Blue (SK-5300, Vector Laboratories Inc., Burlingame, CA, USA). To remove the first primary antibody, sections were cooked in citrate buffer and then washed with PBS. Incubation with the second primary antibody was performed for 1 h in antibody diluent (VWR International, Radnor, PA, USA) at room temperature. Sections were processed with Brightvision kit as described in the preceding text or with poly-AP-anti-rabbit for 30 min at room temperature. Staining was performed using 3'-amino 9'-ethylcarbazole (AEC, Sigma-Aldrich, St. Louis, MO, USA) substrate solution for HRP based detection (HO-1) and alkaline phosphatase substrate kit I Vector Red (SK-5100; Vector Laboratories Inc., Burlingame, CA, USA) for AP based detection (γH2A.X). Sections incubated without the primary antibody were essentially blank. For immunofluorescent labelling, sections were incubated with the respective primary antibodies overnight in antibody diluent (VWR International, Radnor, PA, USA) at 4 °C. The next day, sections were washed with PBS and incubated with Alexa Fluor 568 goat anti-rabbit or Alexa Fluor 488 donkey anti-mouse antibody (Invitrogen, Eugene, OR, USA, 1:200) plus Hoechst 33258 (1:1,000; Thermo Fisher Scientific, Waltham, MA, USA) in antibody diluent (VWR International, Radnor, PA, USA) for 2h at room temperature, mounted with Vectashield (Vector Laboratories Inc., Burlingame, CA, USA) and visualized using Leica Confocal Microscope TCS SP8 X DLS (Leica, Son, the Netherlands) at 20x magnification (bidirectional X, speed 600 Hz, pinhole 1.00 AU).

**Quantification of immunohistochemistry**

Semi-quantitative analysis of surgically resected FCD IIb and TSC tissue was performed as described previously (Arena *et al.,* *Brain Path.*, 2018). Briefly, tissue sections selected for investigation were evaluated by two independent observers for immunoreactivity (IR) using a scale 0-3 (0=absent, 1=weak, 2=moderate, 3=strong staining). All areas of the lesion were examined and the score represents the predominant intensity found in each case. In addition the number of cells positive for the investigated markers was evaluated (0=absent, 1=rare, 2=sparse, 3=high). The product of intensity and number scores was taken to give the overall immunoreactivity score (IRS, Tab. 1).

For *Tsc1*^GFAP-/-^ mouse tissue photomicrographs of whole hippocampus and the sensory-motor cortex were taken at 100x magnification and analysed using ImageJ (v. 1.51) . For 4-HNE images were de-convoluted into DAB and haematoxylin channels and optical density of the DAB area of interest was measured. For HO-1 expression, cells were counted manually on both hemispheres.

**In situ hybridization**

Paraffin-embedded brain tissue was deparaffinised and underwent antigen retrieval as described in the previous paragraph. The oligonucleotide probe for miR155 contained LNA modification, 2-o-methyl modification and digoxygenin (DIG) label (RiboTask ApS, Odense, Denmark). Sections were incubated with the probe (1:750) in hybridization mix (600 mM NaCl, 10 mM HEPES, 1 mM EDTA, 5x Denhardts, 50% Formamide) for 1 h at 56 °C. Sections were washed with saline-sodium citrate (SSC) for 2 min, 0.5x SSC for 2 min and 0.2x SSC for 1 min. After washing with sterile PBS, sections were blocked for 15 min with 1 % bovine serum albumin, 0.02 % Tween 20 and 1 % normal goat serum. Hybridization was detected with AP labelled with anti-DIG (Roche Applied Science, Basel, Switzerland). Nitro-blue tetrazolium chloride (NBT)/5-bromo-4-chloro-3-indolyl phosphate p-toluidine salt (BCIP) was used as chromogenic substrate for AP (1:50 diluted in NTM-T buffer (100 mM Tris, pH 9.5; 100 mM NaCl; 50 mM MgCl2; 0.05 % Tween 20). Thereafter, slides were incubated with antibodies for double-labelling as described in the previous paragraph and visualized using AEC substrate. Negative control assays were performed without probes (sections were blank).

**Western blot analysis**

For protein analysis, cells were washed 2x with PBS and harvested by scraping in lysis buffer containing 10 mM Tris (pH 8.0), 150 mM NaCl, 10 % glycerol, 1 % NP-40, 0.4 mg/ml sodium orthovanadate, 5 mM EDTA (pH 8.0), 5 mM NaF, and protease inhibitors (cocktail tablets, Roche Diagnostics, Mannheim, Germany) and homogenized by pottering and subsequent pipetting. Human tissue samples were homogenized in lysis buffer by pottering. Protein concentrations were estimated by bicinchoninic acid assay (BCA) method (Sigma-Aldrich, Steinheim, Germany). Subsequently, nuclear protein extract (20 µg) was boiled at 100 °C for 5 min. The lysate was then separated using sodium dodecyl sulphate polyacrylamide gel electrophoresis and electrotransferred for 90 min at 100 V to polyvinylidene difluoride (PVDF) membranes (Bio-Rad, Hercules, CA, USA) and then blocked with 5 % skim milk in tris-buffered saline with 0.1% Tween (TBS-T) for 1 h at room temperature. Membranes were cut with a microtome and incubated with the primary antibodies overnight at 4 °C in 5 % skim milk in TBS-T and subsequently washed 3x 10 min in TBS-T. This was followed by incubation with horseradish peroxidase coupled secondary antibodies for 1 h at room temperature in 5 % skim milk in TBS-T. After three washes the membranes were incubated with the SuperSignal West Pico chemiluminescence substrate (ThermoFisher Scientific, Waltham, MA, USA) for 1 min. The blots were then scanned using a LAS-4000 imager (Fujifilm, Tokyo, Japan). Blots were probed with antibodies against ferritin, HO-1, ferroportin 1 (SLC40A1 referred to as FP-1) (rabbit polyclonal, Novus Biologicals, Abingdon, UK; 1:1,000) β-actin (mouse monoclonal, clone C4, Millipore, Darmstadt, Germany; 1:5,000), anti-mouse/HRP (SouthernBiotech, Birmingham, AL, USA; 1:2,500) or anti-rabbit/HRP (Agilent Technologies, Middelburg, the Netherlands; 1:2,500). To quantify the blots, band intensities of individual proteins were measured densitometrically using ImageJ (v. 1.51) and normalized to the loading control β-actin.

**RNA isolation & quantitative real-time PCR**

For RNA isolation, human and mouse tissues as well as cell culture material was homogenized in 700 µL Qiazol Lysis Reagent (Qiagen Benelux, Venlo, the Netherlands). Total RNA, including the miRNA fraction, was isolated using the miRNeasy Mini kit (Qiagen Benelux, Venlo, the Netherlands) according to the manufacturer’s instructions. The concentration and purity of RNA was determined at 260/280 nm using a Nanodrop spectrophotometer (Thermo Fisher Scientific, Wilmington, DE, USA). To evaluate mRNA expression, 250 ng of cell culture derived total RNA or 500 ng tissue-derived total RNA were reverse-transcribed into cDNA using oligo-dT primers. miRNA expression was analysed using miR155 Taqman micro-RNA assays (Applied Biosystems, Foster City, CA, USA). cDNA was generated using Taqman MicroRNA reverse transcription kit (Applied Biosystems, Foster City, CA, USA) according to the manufacturer’s instructions. PCRs were run on a Roche Lightcycler 480 thermocycler (Roche Applied Science, Basel, Switzerland) using reference genes U6 small nuclear RNA (*U6*) for human and small nucleolar 234 (*sno234*) for mouse miRNAs. For mRNA, chromosome 1 open reading frame 43 (*C1ORF43*) and elongation factor 1-α (*EF1-α*) for human mRNA and hypoxanthine phosphoribosyltransferase 1 (*HPRT*) and TATA-Box binding protein (*TBP*) for mouse mRNA were used (see Supp. Tab. 3 for primer sequences). Quantification of data was performed using LinRegPCR as described elsewhere (1).

**Cell cultures**

Primary foetal astrocyte-enriched cell cultures were obtained from human foetal brain tissue (cortex, 14–19 gestational weeks) from medically induced abortions. All material was collected from donors from whom written informed consent for the use of the material for research purposes was obtained by the Bloemenhovekliniek (Heemstede, the Netherlands) . Tissue was obtained in accordance with the Declaration of Helsinki and the Amsterdam UMC Research Code provided by the Medical Ethics Committee. Cell isolation was performed as described previously (1). Briefly, large blood vessels were removed, after which the tissue was mechanically minced into smaller fragments. Tissue was enzymatically digested by incubating at 37 °C for 30 min with 2.5 % trypsin (Sigma-Aldrich, St. Louis, MO, USA). Tissue was washed with incubation medium containing Dulbecco’s modified Eagle’s medium (DMEM)/HAM F10 (1:1) medium (Thermo Fisher Scientific, Waltham, MA, USA), supplemented with 100 units/mL penicillin, 100 µg/mL streptomycin, 1 % glutamine (Thermo Fisher Scientific, Waltham, MA, USA) and 10 % foetal calf serum (FCS; Thermo Fisher Scientific, Waltham, MA, USA) and triturated by passing through a 70 µm mesh filter. Cell suspension was incubated at 37 °C, 5% CO_2_ for 48 h to let glial cells adhere to the culture flask before it was washed with PBS to remove excess of myelin and cell debris. Cultures were subsequently refreshed twice a week. Cultures reached confluence after 2–3 weeks.

TSC cultures were derived from surgical brain tissue (cortex) obtained from patients undergoing epilepsy surgery at the Wilhelmina Children’s Hospital of the University Medical Centre Utrecht (UMCU, Utrecht, the Netherlands). All cases were reviewed and diagnosed as described in the preceding text. Cultures were established and cultured in the same manner as foetal astrocyte-enriched cultures.

Cell cultures for experiments were obtained by trypsinizing confluent cultures and sub-plating onto 96-well (1x10^4^ cells/well for MTT assay) and poly-L-lysine (PLL, 15µg/mL, Sigma-Aldrich, St. Louis, MO, USA)-precoated 6- and 12-well plates (Greiner Bio-One, Kremsmünster, Austria; 2x10^5^ cells/well for Western Blot analysis, 5x10^4^ cells/well for RNA isolation and PCR, 5x10^4^ cells/well with coverslips for immunocytochemistry). Astrocyte-enriched and TSC-derived astrocyte cultures were used at passage 2-5 for all experiments.

**Immunocytochemistry**

Immunocytochemistry on foetal astrocyte-enriched cultures was performed as described previously (2) using γH2A.X primary antibody. The following secondary antibodies or counterstaining were used: Alexa Fluor 488 donkey-anti-mouse antibody (Invitrogen, Eugene, OR, USA; 1:200), Alexa Fluor 594 Phalloidin (Thermo Fisher Scientific, Waltham, MA, USA; 1:200). Coverslips were mounted with Vectashield with DAPI (H-1200, Vector Laboratories Inc., Burlingame, CA, USA). Fluorescent microscopy was performed using Leica Confocal Microscope TCS SP-8X (Leica, Son, The Netherlands) at 200x magnification (bidirectional X, speed 600 Hz, pinhole 1.00 AU). DNA damage was defined by a cutoff of >10 γH2A.X foci in the nucleus of primary foetal astrocytes according to (3). The number of nuclei with >10 γH2A.X foci was counted from at least 15 non-overlapping images and quantified relative to the total number of nuclei as determined by DAPI.

**Analysis of RNA-Sequencing data**

RNA-Sequencing (RNA-Seq) data from 12 TSC cortical tubers and 9 controls analysed by our laboratory in a previous report were utilized in this study (4). Library preparation, quality control, sequence alignment and transcript assembly were performed as described previously. The alignment files for each sample and the unified transcript assembly catalogue were passed to Cuffnorm (5). Using Cuffnorm samples were normalized using the quartile method and an expression matrix containing the Fragments Per Kilobase Million (FPKM) for each gene in each sample was constructed. Following this a log2(FPKM+1) transformation of the expression matrix was carried out. Next, the transformed values for catalase (*CAT*), *GCLC*, glutamate- cysteine ligase modifier subunit (*GCLM*), glutathione peroxidase 1 (*GPX1*), *HMOX1 (HO-1)*, superoxide dismutase 1 (*SOD1*), *SOD2*, ceruloplasmin (*CP*), ferritin heavy chain 1 (*FTH1*), ferritin light chain (*FTL*), glutathione peroxidase 4 (*GPX4)*, lactotransferrin (*LTF*), *SLC7A11* (*xCT*), STEAP3 metalloreductase (*STEAP3*) and transferrin (*TF*) were extracted and statistical analysis was carried out.

**Supplementary tables**

**Supp. Table (1): Clinical information of FCD IIb, TSC and autopsy control cases.**

| **Pathology** | **Gender** | **Age (years)** | **Duration epilepsy** | **Seizure types** | **Brain area** | **Seizures/month** | **Mutation** | **mTOR inhibitors** | **AEDs** |
| --- | --- | --- | --- | --- | --- | --- | --- | --- | --- |
| TSC^a^ | m | 8 mo | 5m | FS | F | >50 | TSC1 | no | VGB |
| TSC^a^ | m | 2 y | 1y | IS | F | unknown | TSC2 | no | VGB |
| TSC^a^ | f | 4 y | 3y | FS, FB/TC | F | >50 | TSC2 | no | VGB |
| TSC^a^ | m | 2 y | 2y | IS | F | unknown | TSC2 | no | VGB, CLB |
| TSC^a^ | f | 2 y | 15m | GS | T | >50 | TSC2 | no | VGB |
| TSC^a^ | f | 6 y | 4y | FS | F | >50 | TSC1 | no | VGB |
| TSC^a^ | m | 1 y | 1y | IS | F | unknown | TSC2 | no | VGB, CLB |
| TSC^b^ | m | 8 y | 7 y | FS, GS | F | 50-100 | TSC2 | no | VGB, CLB |
| TSC^a^ | f | 3 y | 2.5y | FS, FB/TC | T | >50 | TSC2 | no | VGB |
| TSC^a^ | m | 3 y | 2.7y | FS | F | 120 | TSC2 | no | VGB |
| TSC^a^ | m | 8 mo | 8 m | FS | F | 61 | TSC2 | no | VGB, LEV, CLB |
| TSC^b^ | f | 4 y | 10m | FS | F | 600 | TSC1 | no | PHB, CLB, LEV, VGB, OXC, ZNS |
| TSC^b^ | m | 2 y | 2y | FS | F | 600 | TSC2 | no | LEV, VGB |
| TSC^b^ | f | 13 y | 13y | FS | F | 84 | TSC2 | no | LMT, CBZ, CLB |
| TSC^b^ | m | 3 y | 3y | FS | T | 128 | TSC2 | no | TPM, LEV, VPA |
| Fetal TSC | f | 32 GW | - | - | - | - | TSC2 | - | - |
| Fetal TSC | - | 27 GW | - | - | - | - | TSC2 | - | - |
| FCD IIb^a, b^ | m | 41 | 40y | FS, FB/TC | F | 122 | - | - | CBZ, PGB, TPM, CLB |
| FCD IIb^a^ | f | 9 | 6 y | FS, FB/TC | F | 9 | - | - | OXC |
| FCD IIb^a^ | m | 14 | 13y | FS | F | 457 | - | - | VPA, PHT, LMT |
| FCD IIb^a^ | m | 5 | 2y | FS, SE | F | 152 | - | - | VPA, VGB, PHT |
| FCD IIb^a^ | f | 28 | 25y | FS, SE | F | 304 | - | - | PHT, CBZ, LMT |
| FCD IIb^a^ | m | 14 | 10y | FS | F | 213 | n.d. | - | LEV, LMT, CBZ |
| FCD IIb^a^ | m | 21 | 14y | FS, FB/TC | F | 122 | n.d. | - | LEV, LCS, OXC, CNP |
| FCD IIb^a^ | m | 17 | 14y | FS | F | 122 | mTOR | - | LMT |
| FCD IIb^b^ | f | 23 y | 22y | FB, FB/TC | F | 40 | - | - | OXC, LMT |
| FCD IIb^b^ | f | 37 y | 30y | FS, FB/TC | O | 20 | - | - | CBZ, PHT, CLB |
| FCD IIb^b^ | m | 21 y | 15y | FS | F | 450 | - | - | OXC, PHT |
| FCD IIb^b^ | f | 45 y | 19y | FS | F | 375 | - | - | OXC, LMT, CNP |
| Control | m | 3.6 mo | - | - | F | - | - | - | - |
| Control | f | 1 | - | - | T | - | - | - | - |
| Control | f | 2 | - | - | T | - | - | - | - |
| Control | m | 6 w | - | - | F | - | - | - | - |
| Control | f | 7 w | - | - | F | - | - | - | - |
| Control | f | 7 mo | - | - | F | - | - | - | - |
| Control | m | 3 | - | - | F | - | - | - | - |
| Control | m | 4 | - | - | T | - | - | - | - |
| Control | m | 39 | - | - | F | - | - | - | - |
| Control | m | 10 | - | - | F | - | - | - | - |
| Control | f | 17 | - | - | F | - | - | - | - |
| Control | f | 2 | - | - | F | - | - | - | - |
| Control | f | 25 | - | - | F | - | - | - | - |
| Control | m | 13 | - | - | F | - | - | - | - |
| Control | m | 20 | - | - | F | - | - | - | - |
| Control | f | 17 | - | - | F | - | - | - | - |

m = male, f = female, y = year, mo = month, w = weeks, GW = gestational week, SE = status epilepticus, IS = infantile spams, FS = focal seizures, FB/TC = focal bilateral/tonic clonic, F = frontal, T = temporal, O = occipital, CBZ = Carbamazepine, CLB = Clobazam, CNP = Clonazepam, LCS = Lacosamide, LEV = Levetiracetam, LMT = Lamotrigine, PGB = Pregabalin, PHB = Phenobarbital, PHT = Phenytoin, OXC = Oxcarbazepine, TPM =Topiramate, VGB = Vigabatrin, VPA = Valproate, ZNS = Zonisamide, ^a^ = RNA/protein, ^b^ = IHC/ISH

**Supp. Table (2): Oligonucleotide sequence of miR155.**

| **Name** | **Nucleotide sequence** |
| --- | --- |
| miR-155-5p antagomiR | 5’DIG- lAmCmClCmCmUlAmUmClAmCmGlAmUmUlAmGmClAmUmUmAlA-DIG |
| hsa-miR-155-5p mimic | UUAAUGCUAAUCGUGAUAGGGGU |

**Supp. Table (3): Primer sequences used for quantitative real-time PCR**

| **Species** | **Gene** | **Forward primer** | **Reverse primer** |
| --- | --- | --- | --- |
| human | *HO-1* | GGCCAGCAACAAAGTGCAAG | AGTGTAAGGACCCATCGGAGA |
|  | *xCT* | TGACTGGAGTCCCTGCGTAT | TCTTCTTCTGGTACAACTTCCAGT |
|  | *Bach-1* | GCCCAGGACTCCCTTTGTTG | GTTGTCGGGAAGTTCAGTGGA |
|  | *TLR-4* | AATCCCCTGAGGCATTTAGG | AAACTCTGGATGGGGTTTCC |
|  | *TAB-2* | GACAGCACCAGCTCAAGTTC | TGTTCCTTTGCTGCCTAAGTG |
|  | *MYD88* | CTGCTCGAGCTGCTTACCA | CTTCAAGATATACTTTTGGCAATCC |
|  | *FTH-1* | GTGCGCCAGAACTACCACCA | ACATCATCGCGGTCAAAGTAGT |
|  | *FTL* | CTGGAGAAAAAGCTGAACCAG | TCCAGGAAGTCACAGAGATGG |
|  | *TF* | CGAGTCCGACTGTGCTCG | ACTGCACACCATCTCACAGTT |
|  | *CP* | ACATGTGGCCTGACACAGAG | GGTGGAATCCTCAGACTGCC |
|  | *FP-1* | GTGGATCCTTGGCCGACTAC | AAGTGCCACATCCGATCTCC |
|  | *C1ORF43* | GATTTCCCTGGGTTTCCAGT | ATTCGACTCTCCAGGGTTCA |
|  | *EF1α* | ATCCACCTTTGGGTCGCTTT | CCGCAACTGTCTGTCTCATATCAC |
| mouse | *HO-1* | CCTCACAGATGGCGTCACTT | GCTGATCTGGGGTTTCCCTC |
|  | *FTH-1* | CAGAACTACCACCAGGACGC | AGCCACATCATCTCGGTCAA |
|  | *FP-1* | GTCTCTGTCAGCCTGCTGTT | CTTGCAGCAACTGTGTCACC |
|  | *TF* | AGAACCGCTGGTTGGAACAT | GCGCAGCCTTGACTGAAAAA |
|  | *CP* | ATGCTGGGATGGCAACTACC | GGATGTTCCAGGTCATCCTGT |
|  | *HPRT-1* | ATCACATTGTGGCCCTCTG | GTCATGGGAATGGATCTATCACT |
|  | *TBP* | GATGGGAATTCCAGGAGTCA | GAGAATCATGGACCAGAACA |

**Supplementary figures**

**
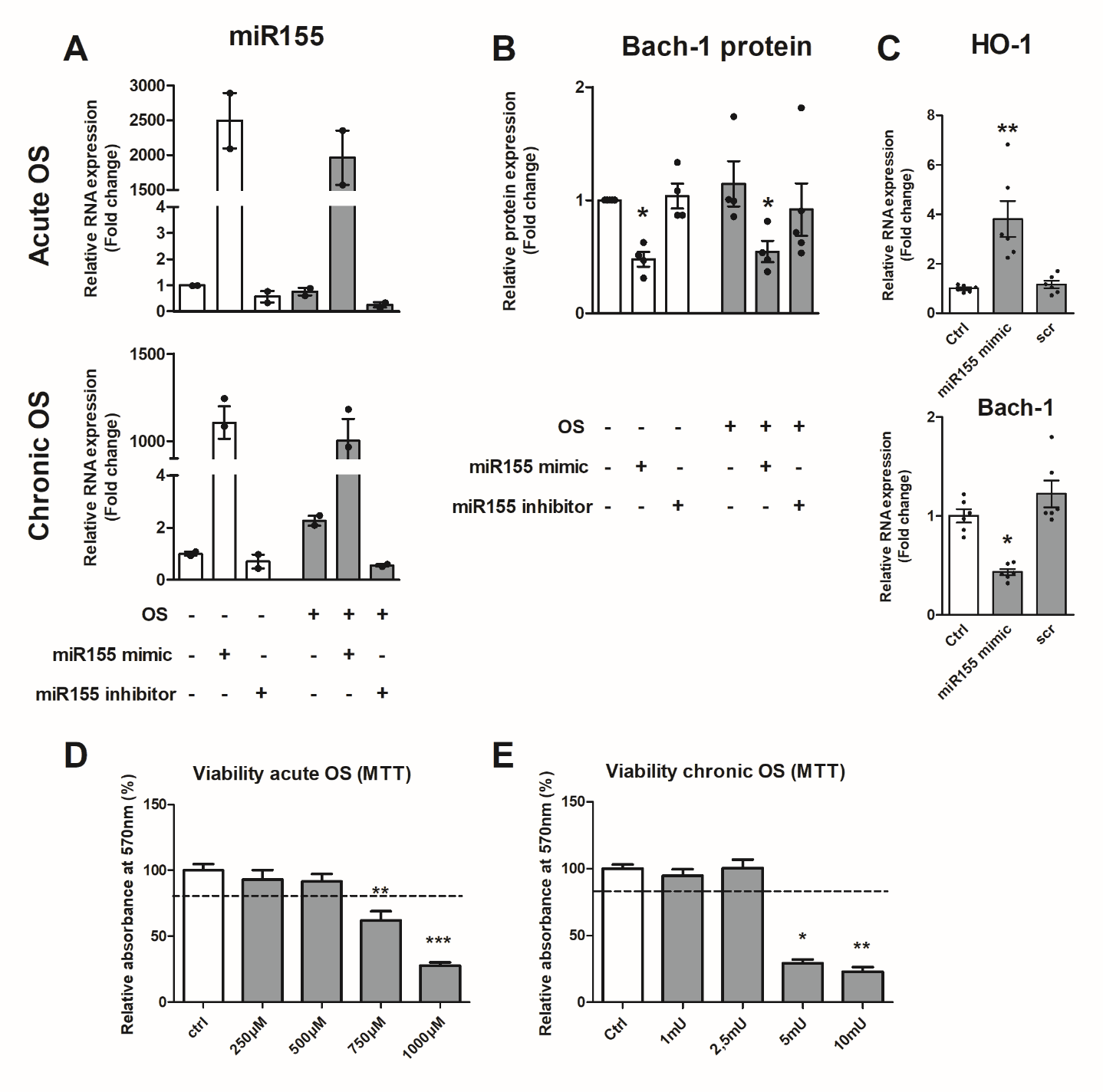
**

**Suppl. Fig. 1 (A)** The expression of miR155 after transfection in human fetal astrocytes was only marginally lowered after 72 h in chronic OS conditions. **(B)** Bach-1 protein expression was lowered by miR155 in human fetal astrocytes transfected for 24 h. **(C)** Transfection of human fetal astrocytes with scrambled construct did not target Bach-1 to induce HO-1. **(D, E)** Human fetal astrocytes is decreased when exposed to >500 µM H_2_O_2_ (3h) or >2.5 mU GO (24h). Data in A-C presented as mean, error bars represent SEM; * p<0.05, **p<0.01, ***p<0.001. Data are representative of one (A) or three (B-E) independent experiments with two (A-C) or four (D, E) replicates for each group.


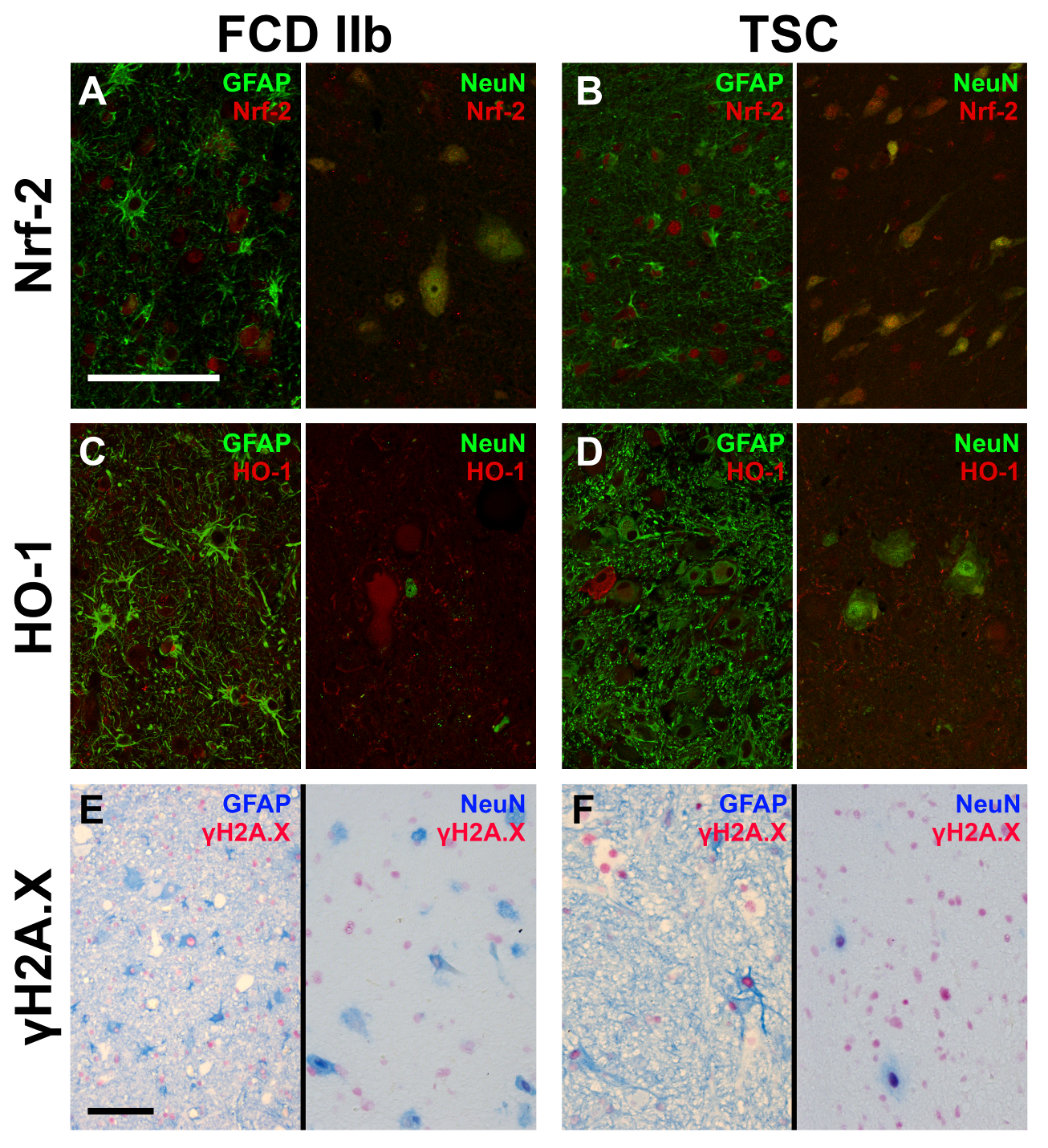


**Suppl. Fig. 2 (A, B)** Double labelling of Nrf-2 with GFAP and NeuN revealed nuclear expression in astrocytes and neurons. **(C, D)** HO-1 expression could be detected primarily in the cytoplasm of astrocytes, and some neurons, as well as in NeuN and GFAP negative cells. **(E, F)** Nuclear expression of γH2A.X could be detected in astrocytes and some neurons. Scale bar 100 µm in A, E.


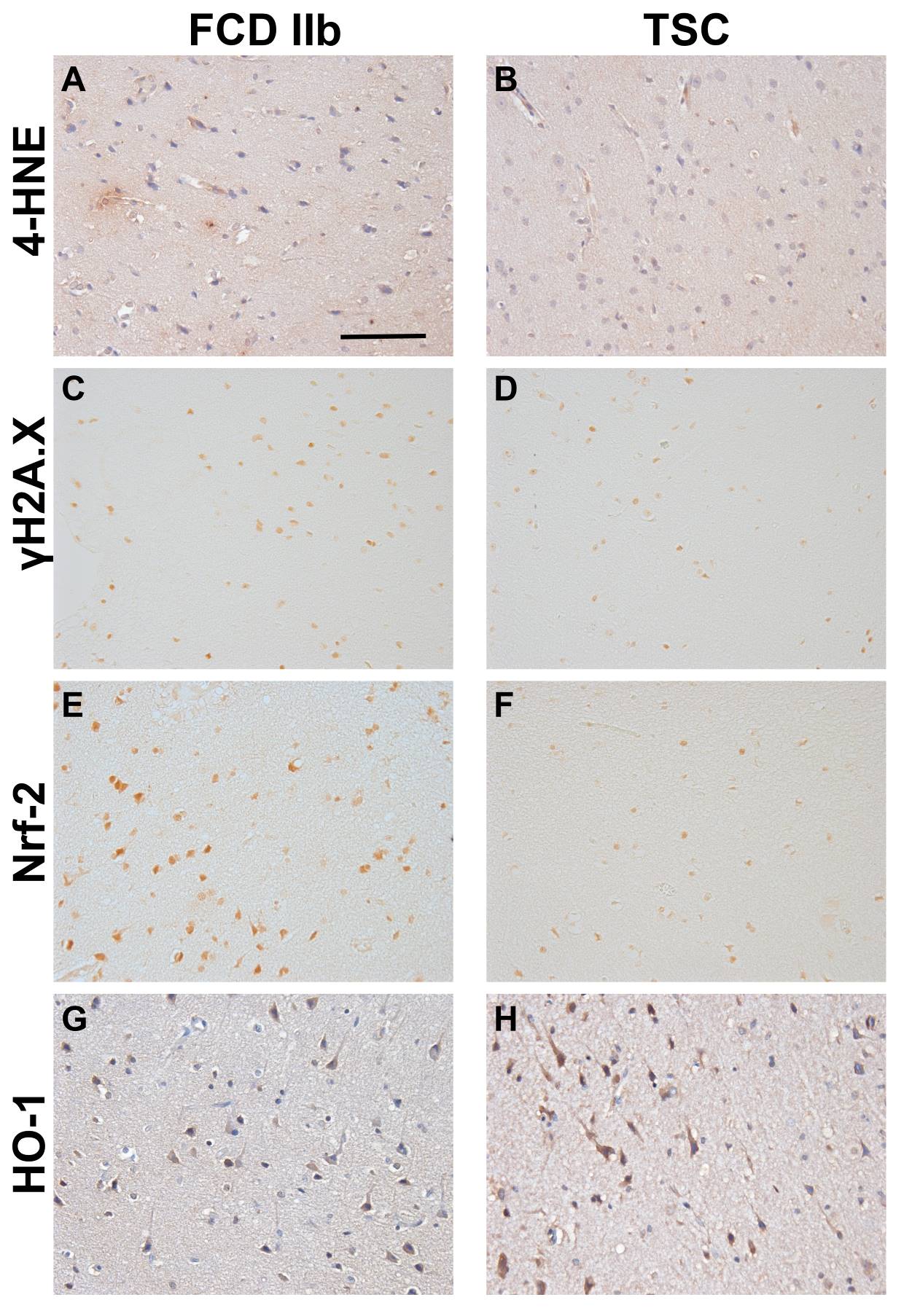


**Suppl. Fig. 3 (A, B)** 4-HNE reactivity in FCD IIb and TSC perilesional tissue was confined to low neuronal and perivascular expression. **(C, D)** γH2A.X expression in perilesional tissue could be detected in all cell types. **(E, F)** Perilesional Nrf-2 expression was expressed in all cell types and higher than in autopsy control tissue. **(G, H)** HO-1 expression in perilesional areas was mainly confined to neurons and some cells with glial morphology. Scale bar 100 µm in A.

1. van Scheppingen J, Iyer AM, Prabowo AS, Muhlebner A, Anink JJ, Scholl T, et al. Expression of microRNAs miR21, miR146a, and miR155 in tuberous sclerosis complex cortical tubers and their regulation in human astrocytes and SEGA-derived cell cultures. Glia. 2016;64(6):1066-82.

2. van Scheppingen J, Broekaart DW, Scholl T, Zuidberg MR, Anink JJ, Spliet WG, et al. Dysregulation of the (immuno)proteasome pathway in malformations of cortical development. J Neuroinflammation. 2016;13(1):202.

3. Bahjat M, de Wilde G, van Dam T, Maas C, Bloedjes T, Bende RJ, et al. The NEDD8-activating enzyme inhibitor MLN4924 induces DNA damage in Ph+ leukemia and sensitizes for ABL kinase inhibitors. Cell Cycle. 2019;18(18):2307-22.

4. Mills JD, Iyer AM, van Scheppingen J, Bongaarts A, Anink JJ, Janssen B, et al. Coding and small non-coding transcriptional landscape of tuberous sclerosis complex cortical tubers: implications for pathophysiology and treatment. Sci Rep. 2017;7(1):8089.

5. Trapnell C, Hendrickson DG, Sauvageau M, Goff L, Rinn JL, Pachter L. Differential analysis of gene regulation at transcript resolution with RNA-seq. Nat Biotechnol. 2013;31(1):46-53.
